# MEDUSA: A Pipeline for Sensitive Taxonomic Classification and Flexible Functional Annotation of Metagenomic Shotgun Sequences

**DOI:** 10.3389/fgene.2022.814437

**Published:** 2022-03-07

**Authors:** Diego A. A. Morais, João V. F. Cavalcante, Shênia S. Monteiro, Matheus A. B. Pasquali, Rodrigo J. S. Dalmolin

**Affiliations:** ^1^ Bioinformatics Multidisciplinary Environment, Federal University of Rio Grande do Norte, Natal, Brazil; ^2^ Graduate Program in Engineering and Natural Resources Management, Federal University of Campina Grande, Campina Grande, Brazil; ^3^ Academic Food Engineering Unit, Federal University of Campina Grande, Campina Grande, Brazil; ^4^ Department of Biochemistry, Federal University of Rio Grande do Norte, Natal, Brazil

**Keywords:** metagenomics, bioinformatics, taxonomic classification, functional annotation, pipeline, shotgun sequences

## Abstract

Metagenomic studies unravel details about the taxonomic composition and the functions performed by microbial communities. As a complete metagenomic analysis requires different tools for different purposes, the selection and setup of these tools remain challenging. Furthermore, the chosen toolset will affect the accuracy, the formatting, and the functional identifiers reported in the results, impacting the results interpretation and the biological answer obtained. Thus, we surveyed state-of-the-art tools available in the literature, created simulated datasets, and performed benchmarks to design a sensitive and flexible metagenomic analysis pipeline. Here we present MEDUSA, an efficient pipeline to conduct comprehensive metagenomic analyses. It performs preprocessing, assembly, alignment, taxonomic classification, and functional annotation on shotgun data, supporting user-built dictionaries to transfer annotations to any functional identifier. MEDUSA includes several tools, as fastp, Bowtie2, DIAMOND, Kaiju, MEGAHIT, and a novel tool implemented in Python to transfer annotations to BLAST/DIAMOND alignment results. These tools are installed via Conda, and the workflow is managed by Snakemake, easing the setup and execution. Compared with MEGAN 6 Community Edition, MEDUSA correctly identifies more species, especially the less abundant, and is more suited for functional analysis using Gene Ontology identifiers.

## 1 Introduction

The recent reduction of sequencing costs, a consequence of second-generation sequencing technology advances, notably benefited the metagenomics field. Metagenome shotgun sequencing became widely used, allowing microbial DNA sequencing from an environmental sample without selecting any particular gene. The taxonomic classification of environmental DNA provides species composition information for biodiversity studies (Pedersen et al., 2015). Shotgun data also contains information about the microbial community functional activity, adding ecological information to metagenomic studies.

There are two metagenomic analysis approaches: read classification and metagenomic assembly (Breitwieser et al., 2019). These approaches share common analysis steps, such as data preprocessing, the alignment against a reference database, taxonomic classification, and functional annotation. The difference is a step to assemble reads into contigs, after the preprocessing, on the assembly approach. The choice between direct read classification and assembly-based analysis depends on the analysis goal and research question. Read classification is useful for organisms with close relatives represented in the reference database. For samples collected from exotic environments, when no close relatives are expected to be found in the reference database, the assembly approach is desirable. But one approach does not exclude the other, and assemblies may be used to support classifications made directly from the reads. There are several tools available for each analysis step, with varying accuracies. Therefore, the toolset choice impacts the analysis results and conclusions (Lindgreen et al., 2016), and efficiently selecting a toolset to conduct a complete metagenomics analysis remains challenging.

Some tools are well established, such as the DIAMOND aligner (Buchfink et al., 2015), which stands out for its speed and accuracy. Hence, this aligner is commonly used in pipelines and tools for metagenomics and metatranscriptomics, such as SAMSA2 (Westreich et al., 2018), MetaErg (Dong and Strous, 2019), HUMAnN2 (Franzosa et al., 2018), eggNOG-mapper (Huerta-Cepas et al., 2017), and GO FEAT (Araujo et al., 2018). The DIAMOND aligner performs protein alignments, a compute-intensive task that produces a functional result with protein identifiers (IDs) according to the database used as reference.

As a consequence, these tools and pipelines for metagenomic analysis present results with specific identifiers. GO FEAT reports Gene Ontology (GO) identifiers in its results, and eggnog-mapper reports Orthologous Groups identifiers. Even using DIAMOND for the alignments in both tools, the different reference databases used for each one make the intermediate files not exchangeable. Thus, the alignment must be performed for each tool separately to get the two types of identifiers. To ease multiple executions, some pipelines for metagenomic shotgun sequences analysis are fully automated, such as Sunbeam (Clarke et al., 2019) and MetaErg. Sunbeam, for example, adopts the use of the Snakemake workflow management software (Köster and Rahmann, 2012) to achieve reproducibility and automation.

MEGAN 6 is a software widely used for microbiome analysis that translates protein IDs into others, such as GO and InterPro, using SQLite databases. MEGAN is available in two versions, the Community Edition (CE) (Huson et al., 2016) and the Ultimate Edition (UE). The CE is freely available and allows the download of an SQLite dictionary mapping NCBI-nr (National Center for Biotechnology Information—non-redundant) accessions to taxonomy, eggNOG, and a mix of InterPro and GO IDs. Whereas the UE requires an annual license and includes mappings for KEGG, SEED, RDP, and Pfam IDs.

The selection of tools suited for each step of a metagenomic analysis is a challenge. The standalone pipelines available in the metagenomics field produce results containing a specific set of functional identifiers, narrowing the capabilities to extract insights beyond the scope of the identifier type reported. Web-based pipelines might restrain access to intermediate files, useful to conduct other analyses, and the fine-tuning of tool’s parameters to achieve a better result. We aim with this work to address these presented issues by surveying tools from the literature and benchmarking them to design a fully automated analysis pipeline that allows functional annotation transfer through user-built identifier mapping dictionaries.

Here we introduce a new pipeline for metagenomic analyses. The MEDUSA pipeline performs steps for both metagenomic approaches, accurate and sensitive taxonomic classifications, and functional annotations using fast disk storage repositories created from plain text dictionaries. The whole pipeline is available as an environment at the Anaconda Cloud, easing software acquisition and setup via the Conda package manager. Installing and running details can be found in the Supplementary Material.

## 2 Materials and Methods

### 2.1 Pipeline Overview

**FIGURE 1:**
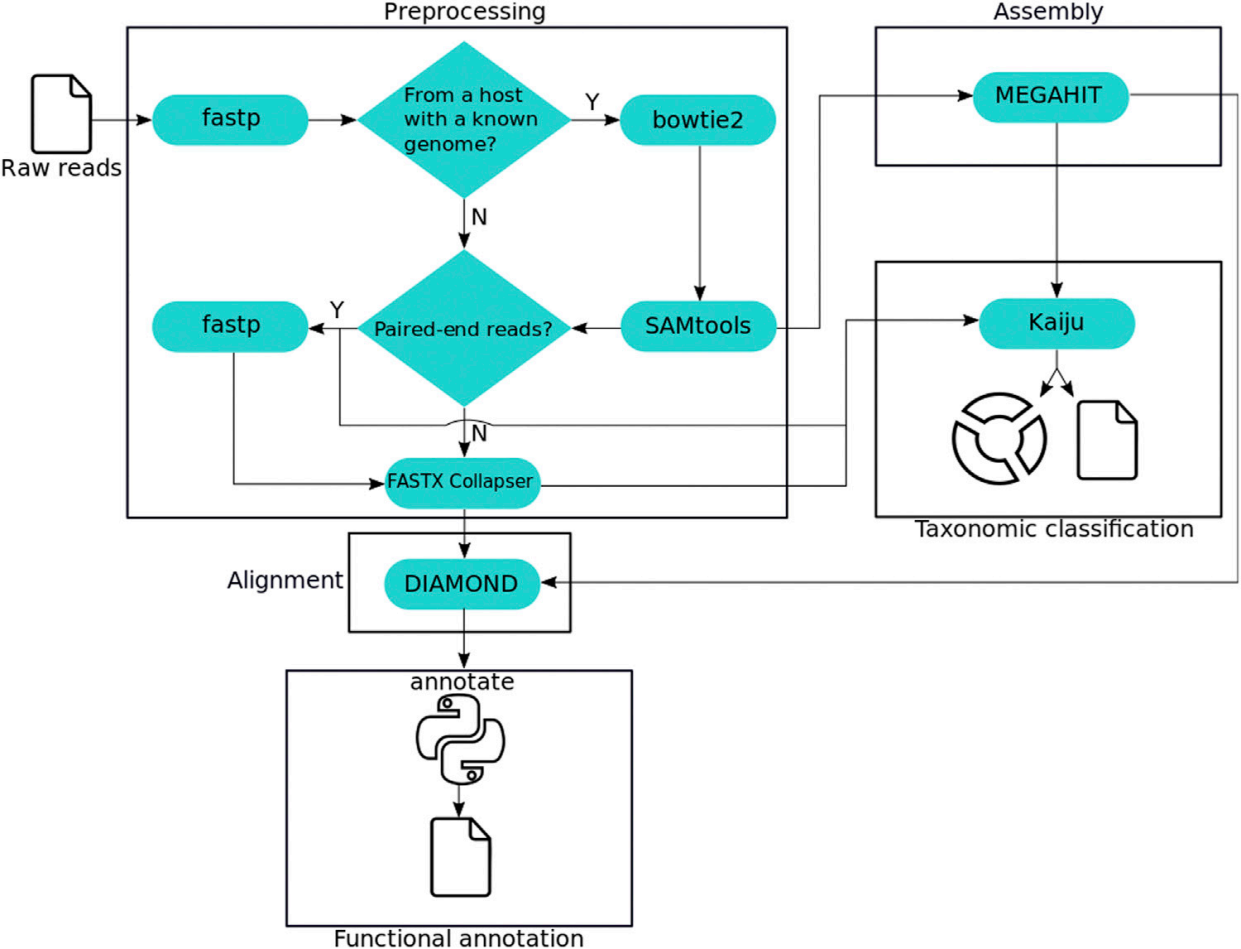
MEDUSA analysis workflow. Squares highlight the protocol steps, and third-party tools are depicted as cyan capsules. The python icon represents the tool implemented for the functional annotation.

We surveyed the literature and selected a set of state-of-the-art tools for each of four analysis steps: preprocessing, alignment, assembly, and taxonomic classification. Fastp was chosen for its speed and features, as the interactive quality control report produced reading the input only once. Bowtie2 for speed and accuracy, obtaining a low misclassification rate. Kaiju for achieving the highest Matthews Correlation Coefficient (MCC) (Chicco and Jurman, 2020) at the species and genus level. Lastly, DIAMOND and MEGAHIT for the performance in published benchmarks. Figure 1 shows the workflow designed to include these tools and perform all steps required for a comprehensive metagenomic analysis.

### 2.2 Datasets

#### 2.2.1 Dataset for Trimming

To assess trimming tools, we downloaded the run SRR5371509 from the Sequence Read Archive (SRA). The original raw reads from this bovine fecal metagenome were split into files containing 1, 5, 10, and 40 million reads in a paired-end (PE) format.

#### 2.2.2 Dataset for Decontamination

To assess decontamination tools, we downloaded a human primary assembly from NCBI (RefSeq NC_000004.12) and used the InSilicoSeq software (Gourlé et al., 2019) to create three simulated datasets. The primary assembly NC_000,004.12 represents the assembled human chromosome 4, used by InSilicoSeq as the source to generate human reads for these simulated datasets. The InSilicoSeq software can download random genomes directly from NCBI. It is possible to choose between bacteria, viruses, archaea, or a combination of these options with the argument --ncbi/-k, as well as the number of genomes with the argument --n_genomes/-u. For this dataset we downloaded 200 bacterial genomes with InSilicoSeq, using --seed 5 to control the random number generation. Each dataset was created with 4 million reads, with human reads composing 25%, 50%, and 75% of the dataset, and bacterial reads generated by InSilicoSeq composing the remaining.

#### 2.2.3 Dataset for Assembly

Critical Assessment of Metagenome Interpretation (CAMI) (Meyer et al., 2021) provides reads and its respective gold-standard assembly (GSA). Three datasets, labeled as low, medium, and high complexity, were created for the first CAMI challenge using the CAMISIM microbial community and metagenome simulator. The low complexity dataset has a small insert size, the medium complexity has differential abundances of respective organisms, and short and long insert sizes, and the high complexity dataset is a time series of samples with a small insert size. The small insert size has 270 bp, and the long has 5,000 bp. To assess the assembly, we downloaded the low, medium, and high complexity datasets from the first CAMI challenge.

#### 2.2.4 Dataset for Taxonomic Classification

Some reads used in metagenome studies end not being assigned to a taxonomic identifier. This outcome means that these reads have no matches among the reference database sequences, and as these reads may be from organisms not present in the reference database, they are labeled as “unknown organisms.” To assess the taxonomic classification, we created a dataset containing 509,688 Illumina MiSeq reads following a lognormal distribution with InSilicoSeq, from which 99,918 are negative control (NC) reads simulating unknown organisms. Although the number of reads to be generated was set to 500,000 and 100,000 (20% of the simulated dataset), respectively, using the --n_reads InSilicoSeq argument, the output presents a slightly different number of reads. The metadata from this dataset can be found in our GitHub repository. This dataset was generated from 394 bacterial, 73 archaeal, and 40 viral sequences, without duplicates, randomly downloaded by InSilicoSeq. To simulate the unknown organisms, NC, we used InSilicoSeq and 199 bacterial sequences, shuffled by the esl-shuffle command from HMMER (HMMER, 2021) v3.3 (http://hmmer.org/) using non-overlapping windows of size 500 (-w 500). In what follows, this dataset will be mentioned as Dataset 1 (D1).

#### 2.2.5 Dataset for Functional Annotation

We selected sequences from 10 bacterial organisms to use as a source to create 400,433 reads with InSilicoSeq. The GenBank identifiers from the selected sequences are described in the Supplementary Material. The UniProt ID mapping API (https://www.uniprot.org/help/api_idmapping) was used to convert the GenBank IDs (EMBL_ID) to UniProt IDs (ACC), allowing to transfer curated UniProtKB/Swiss-Prot GO information from the UniProt IDs to the GenBank IDs. Finally, these sequences were concatenated with the NC created to assess the taxonomic classification. In what follows, this dataset will be mentioned as Dataset 2 (D2).

#### 2.2.6 Public Dataset Selected

We selected a public human gut metagenome shotgun data from a patient with Crohn’s disease (run SRR579292 from the BioProject PRJNA175224). In what follows, this dataset will be mentioned as Dataset 3 (D3).

### 2.3 Benchmarks

As the preprocessing involves different tasks, such as quality control and host sequences removal, two benchmarks were designed to evaluate tools for these purposes.

#### 2.3.1 Trimming Tools Benchmark

The quality control check is performed to identify and remove low-quality reads, and the following tools able to accomplish this task were selected for comparison: AfterQC (Chen et al., 2017), BBDuk (BBTools, 2021) (http://jgi.doe.gov/data-and-tools/bb-tools/), Cutadapt (Martin, 2011), Fastp (Chen et al., 2018a), SOAPnuke (Chen et al., 2018b), and Trimmomatic (Bolger et al., 2014). We applied these tools to the dataset created to assess the trimming performance, processing the PE files, and also only the forward reads, to simulate a single-end (SE) input. The inputs were processed using one and four computing cores to assess the reduction in the elapsed time, an expected consequence of the parallelism. The speed of each tool was measured using the “time” Unix command and averaging three runs. As a reference for the elapsed time, we also ran the FastQC software (Babraham, 2021) (https://www.bioinformatics.babraham.ac.uk/projects/fastqc/). FastQC is a tool used to create visual reports detailing the quality of the reads before and after the preprocessing. As the parallelism supported by FastQC only allows the use of multiple inputs, not reducing the time required for processing one input, it was benchmarked with one computing core.

#### 2.3.2 Decontamination Tools Benchmark

The strategy used for host sequences removal is to use a tool to align the reads against a reference genome, such as the Ensembl Homo sapiens GRCh38 for reads sequenced from humans, and then filter out the aligned sequences. To align reads against a reference genome we selected: BBMap (BBTools, 2021) (http://jgi.doe.gov/data-and-tools/bb-tools/), Bowtie2 (Langmead and Salzberg, 2012), BWA (Li and Durbin, 2009), and HISAT2 (Pertea et al., 2016). We measured the speed of the tools and, as the source used to generate each read from the decontamination dataset is known, the quality of the results using the MCC. The MCC ranges from -1, only false negatives (FN) and false positives (FP) classifications, to 1, a perfect classification with only true negatives (TN) and true positives (TP).

#### 2.3.3 Assembly Tools Benchmark

The assembly step produces contigs, longer DNA sequences resulting from the overlap of reads. Modern assemblers, such as MEGAHIT (Li et al., 2016) and MetaSPAdes (Nurk et al., 2017), use de Bruijn graphs. These assemblers were extensively benchmarked by the CAMI. Thus, researchers might use assemblers and submit the results to CAMI, or assess the results using MetaQUAST (Mikheenko et al., 2016) and the GSA. To benchmark MEGAHIT and MetaSPAdes, we used the low, medium, and high complexity datasets from the first CAMI challenge.

#### 2.3.4 Taxonomic Tools Benchmark

It is possible to assign a taxonomy identifier to a read using different approaches, such as the use of *k*-mers or alignments. BASTA (Kahlke and Ralph, 2019) and Krona (Ondov et al., 2011) transfer annotations to alignment results. While Kaiju (Menzel et al., 2016) and Kraken 2 (Wood et al., 2019) perform classifications using reads as inputs. We applied BASTA and Krona to the DIAMOND output resulting from the alignment of this dataset against the NCBI-nr database. DIAMOND, Kraken 2, and Kaiju indices were built using the NCBI-nr as reference database. Krona is mainly used for taxonomic results visualization, but it is possible to use ktClassifyBLAST to assign taxonomy identifiers to BLAST/DIAMOND results. As the annotation transfer performed by Krona is simpler than the performed by BASTA, we used Krona’s MCC as a lower bound reference for taxonomic classifications based on annotation transfer.

### 2.4 Aligner Choice

For the alignment, we selected DIAMOND due to its speed, accuracy, and adoption in several tools and pipelines. As the Bowtie2 output is used as the DIAMOND input, we use SAMtools (Li et al., 2009) to extract the unaligned reads from the Bowtie2 output. As a protein aligner, the DIAMOND output might be used for both taxonomic and functional analyses. Amino acid sequences are more conserved than DNA sequences when taking into account evolutionary distances among sequences. Furthermore, homology searches using a six-frame translation of DNA sequences against protein databases improve taxonomic and functional results. The DIAMOND software performs this task by building a double-index, over the translated reads and the protein database, sorted lexicographically and traversed linearly to determine matching seeds. Seeds are amino acid fragments, varying according to the DIAMOND sensitivity mode used, with more sensitive modes using more seeds on the matchings. The DIAMOND is used in our pipeline to align the sequences after the preprocessing, with the NCBI-nr as the reference protein database. Then, the protein identifiers reported in DIAMOND results are used to get a functional identifier of interest in the functional annotation step.

### 2.5 Annotation Transfer for Functional Results

The DIAMOND output contains functional information, appearing in the results as RefSeq and GenBank IDs due to the use of NCBI-nr as the protein database. To allow the reuse of an alignment output to obtain different functional IDs, we implemented a tool in Python to transfer annotations to BLAST/DIAMOND alignment results. This tool, named *annotate*, creates fast disk storage repositories from custom plain text dictionaries, filter hits according to user-defined thresholds, and assigns functional IDs to the best hit possible from each read. Alignments not meeting thresholds for e-value, bit-score, percent identity, or alignment length, are ignored. If a read contains no alignment passing the thresholds, or none could be mapped, it is assigned to “Unknown”. Furthermore, it is also possible to omit unknown mappings from the output or to map all the alignments. Annotate processes the alignment output linearly, requiring less time and memory than to create a new database and perform a new alignment.

### 2.6 Automating the Analysis and Comparing With MEGAN 6 CE

The pipeline designed after the benchmarks is composed of the tools most suited for each step. The pipeline’s execution rules were detailed using Snakemake, a workflow management system for scalable and reproducible data analyses. To ease software acquisition and setup, we created an environment containing all the pipeline’s tools and dependencies at the Anaconda cloud. Finally, we used three datasets, D1, D2, and D3, to assess the pipeline’s results compared to those obtained by MEGAN v6.18.3 CE. The D1 was the dataset created to benchmark the taxonomic tools. The D2 was created using 10 bacterial sequences as source, with curated functional information. D1 metadata allows assessing the taxonomic results, and D2 assesses the functional results.

We used a phred score threshold of 20 to trim all datasets, the Ensembl Homo sapiens GRCh38 DNA primary assembly to identify host sequences, and the NCBI-nr as the reference database. We preprocessed and aligned the three datasets with the designed pipeline, submitting the outputs to the taxonomic and functional analyses using MEGAN 6 CE and MEDUSA. For MEGAN, the only argument changed was the identity threshold, set to 80%. As the default minimum percent identity threshold used by MEGAN is 0%, we changed it to conduct a fair comparison between both methodologies with more accurate hits. A percent identity threshold above 70% is frequently used for this purpose. We choose 80% to achieve higher accuracies in the results, and we set this value as the default percent identity threshold used by annotate. We created a dictionary for the functional analysis performed by our pipeline, mapping GenBank and RefSeq IDs to GO IDs. It was done using the UniProt ID mapping file and the R programming language (version 4.0.5).

## 3 Results

### 3.1 Trimming Tools Results

**FIGURE 2:**
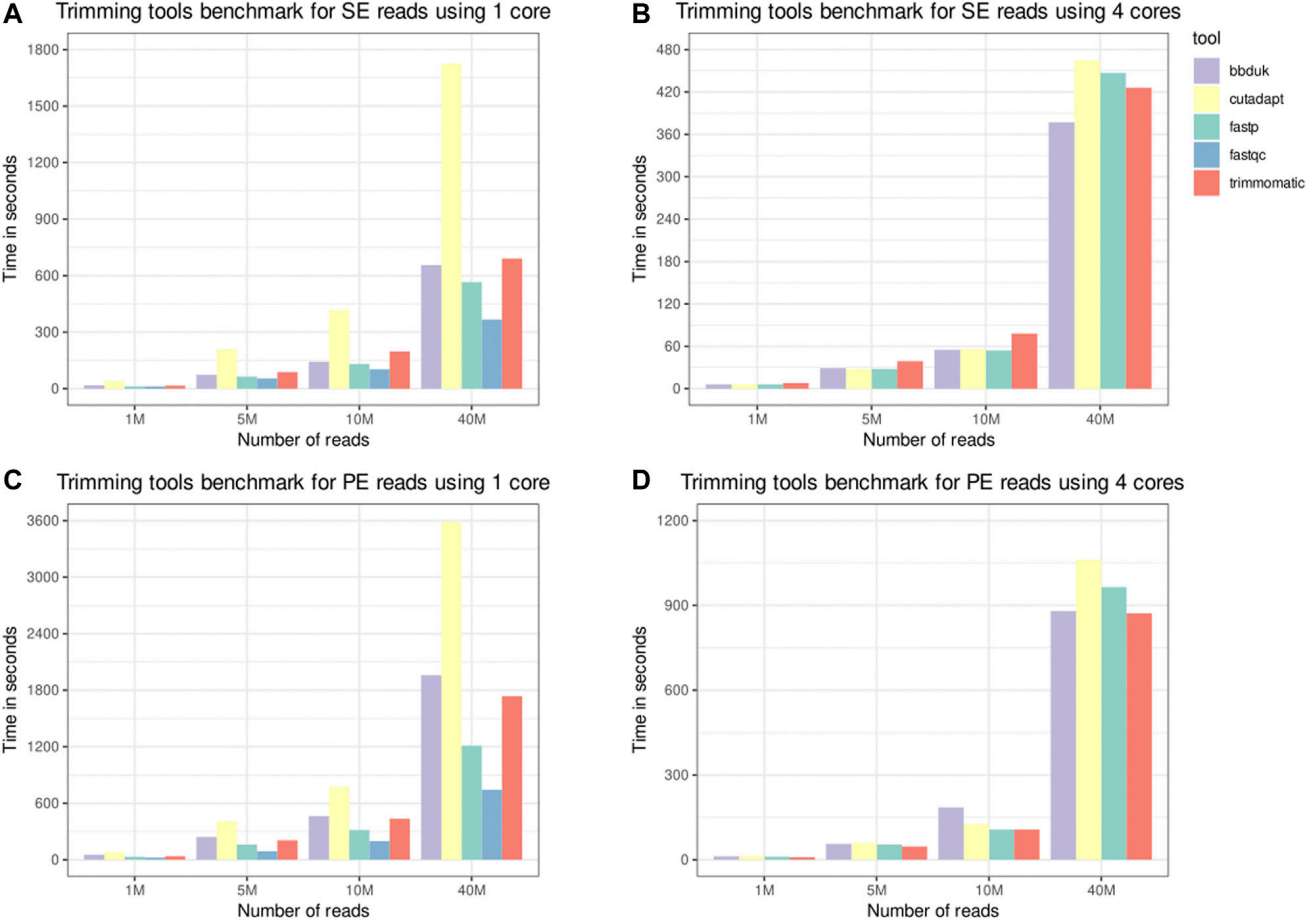
Trimming tools benchmark. Single-end (SE) and paired-end (PE) inputs, containing 1, 5, 10, and 40 million reads, were processed by the selected tools. A phred score threshold of 20 was used for all tools. The “time” Unix command was used to measure the elapsed time, and the times depicted in the panels are the average of three runs. Panels **(A,C)**, respectively, depict the time for SE and PE inputs using only one thread. Panels **(B,D)**, respectively, depict the time for SE and PE inputs using four threads.

The trimming tools benchmark results are depicted in Figure 2. SOAPnuke was removed from the results for presenting outputs with a different number of reads when the only parameter change was the number of cores. AfterQC presented execution times much slower than the other tools, and as Fastp was developed as a faster alternative to it, we discarded AfterQC from the benchmark results. FastQC was benchmarked with only one computing core as its implementation of parallelism does not reduce the processing time for a single file. As expected, FastQC was faster than other tools using one core due to the reduced number of tasks performed. Fastp is the second-fastest tool when only one computing core is used and is fast enough when four computing cores are used. Only Fastp and FastQC produce visual reports, both containing information from before and after the processing. The benefits from producing the report may overcome the low increase in the elapsed time on scenarios with a larger number of reads. As an all-in-one FASTQ preprocessor, Fastp has useful features as PE reads merging and performs more tasks.

### 3.2 Decontamination Tools Results

**FIGURE 3:**
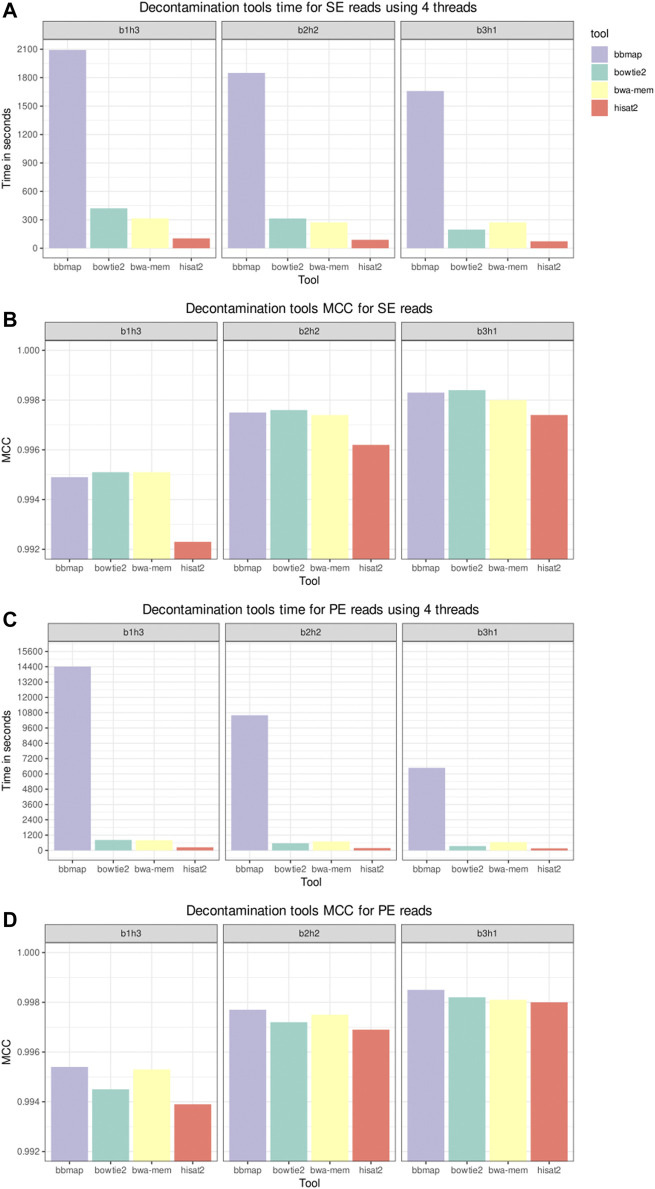
Decontamination tools benchmark for time and Matthews Correlation Coefficient. Single-end (SE) and paired-end (PE) inputs, composed by 25% (b3h1), 50% (b2h2), and 75% (b1h3) of human reads, were processed by the selected tools. The Ensembl Homo sapiens GRCh38 DNA primary assembly version 102 was used as a reference to build the indices. The “time” Unix command was used to measure the elapsed time, and the time depicted in panels **(A,C)** is the average of three runs. Panels **(B,D)**, respectively, depict the Matthews Correlation Coefficient (MCC) for SE and PE inputs.

**FIGURE 4:**
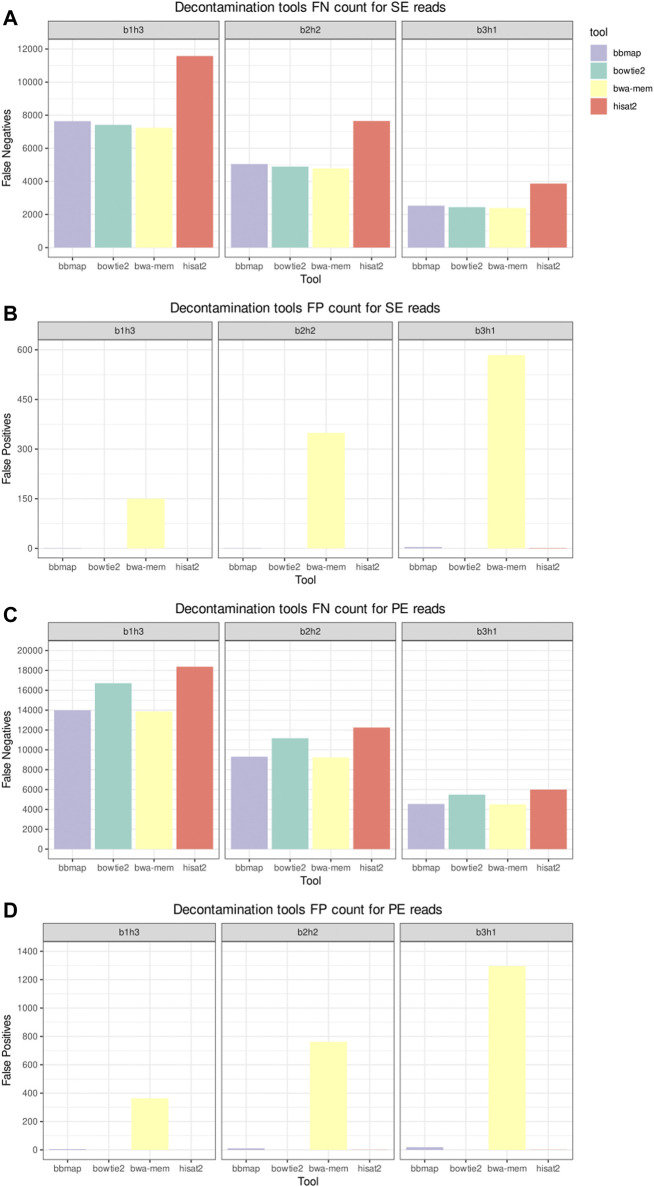
Decontamination tools misclassification benchmark. Panels **(A,C)**, respectively, depict the false negative (FN) counts for the single-end (SE) and paired-end (PE) inputs. Panels **(B,D)**, respectively, depict the false positive (FP) counts for the SE and PE inputs.

Figure 3 shows the elapsed time and MCC from the host sequences removal tools in the decontamination benchmark. The datasets used in this benchmark were labeled according to their composition, with the label b1h3 meaning 25% of bacterial reads and 75% of human reads. BBMap was the slowest tool on all scenarios, and HISAT2 was the fastest. As all tools achieved a high MCC, above 0.99, we inspect the FN and FP counts to distinguish the performances. Figure 4 shows the FN and FP for SE and PE reads. Overall, the BWA-MEM algorithm had more FP, and HISAT2 had more FN. BBMap and Bowtie2 achieved a higher MCC on the scenarios with fewer human reads, being more sensitive than BWA and HISAT2 to detect contaminants on these scenarios.

### 3.3 Assembly Tools Results

In the assembly benchmark, all tests performed with MetaSPAdes failed due to memory-related issues. MetaSPAdes 3.15.2 was unable to allocate the required memory on all runs, and version 3.13 fails due to a segmentation fault right after starting the job. MEGAHIT finished all runs successfully and was chosen to compose the pipeline due to the results from CAMI benchmarks (Meyer et al., 2021).

### 3.4 Taxonomic Tools Results

**FIGURE 5:**
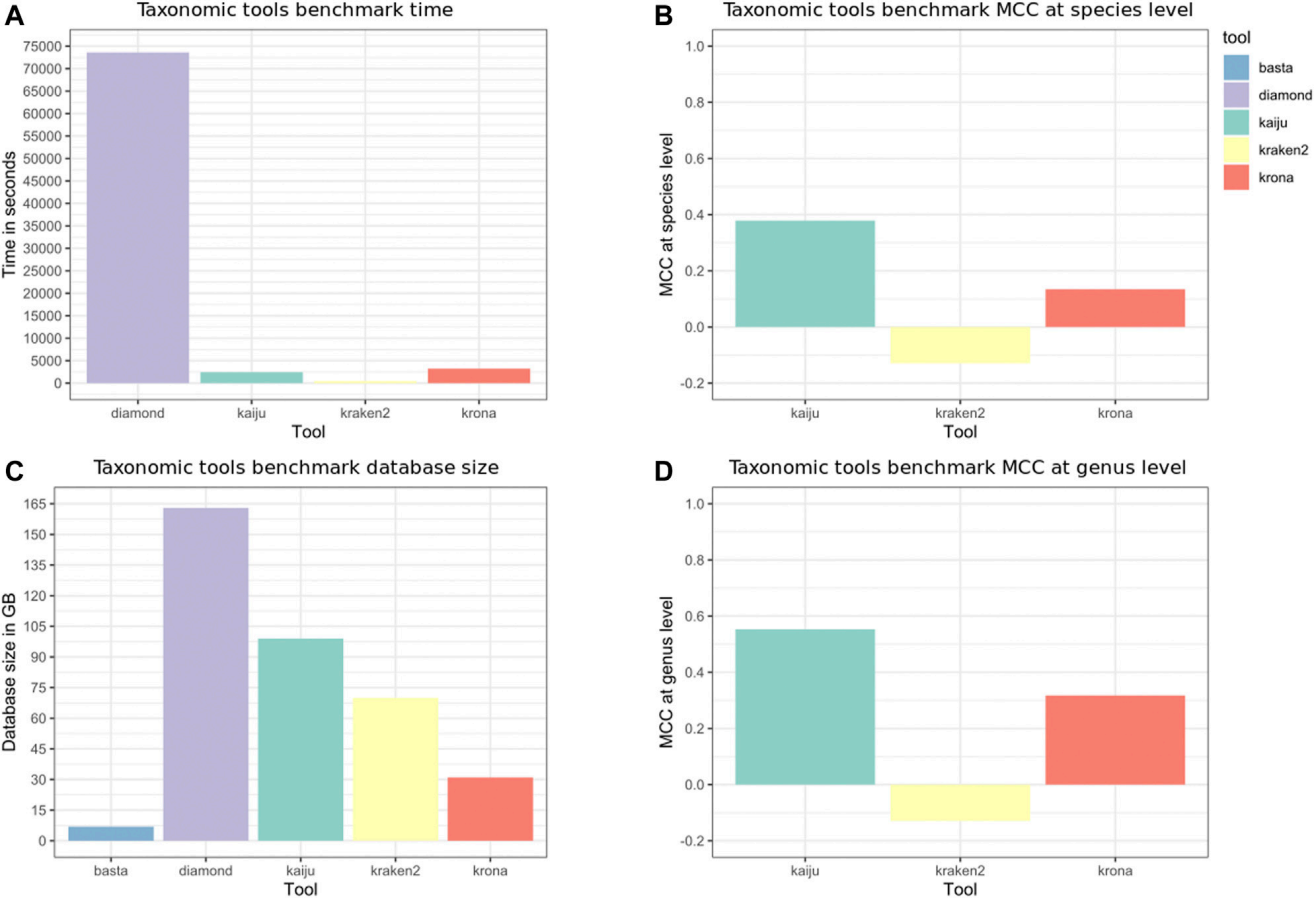
Taxonomic tools benchmark. Krona and BASTA require an alignment output to classify the reads, while Kaiju and Kraken accept Single-end (SE) or paired-end (PE) inputs. DIAMOND was used to align the D1 reads, and the NCBI-nr was used as reference to build the indices and databases. The “time” Unix command was used to measure the elapsed time, and the time depicted in panel **(A)** is the average of three runs, not taking into account the time needed to build the indices and databases. Panel **(C)** depicts the database size in GB, being smaller for transfer annotation tools (Krona and BASTA). Panels **(B,D)**, respectively, depict the Matthews Correlation Coefficient (MCC) at the species and genus level. BASTA is not depicted in panel **(D)** as the classification took more than 20 days.

The taxonomic tools benchmark results are shown in Figure 5. Kraken 2 ran fast and without errors, but classified only 2129 reads from 507429 (0.42%). We built again the Kraken 2 index and noticed that only a few identifiers from NCBI-nr were mapped. We then used the fix_unmapped script from Kraken tools, but although the new index correctly mapped almost all identifiers, no reads were classified (0%). For the tools that require alignment results, such as Krona and BASTA, we need to consider the time spent to align the reads. BASTA requires less space on the disk to store the databases needed for the analyses, but the runs took more than 20 days and were aborted. Comparing the classifications performed by Krona and Kaiju, Kaiju achieved better performance at species and genus level and runs faster as processes the reads, accepting SE and PE inputs.

### 3.5 Comparison Results

**FIGURE 6:**
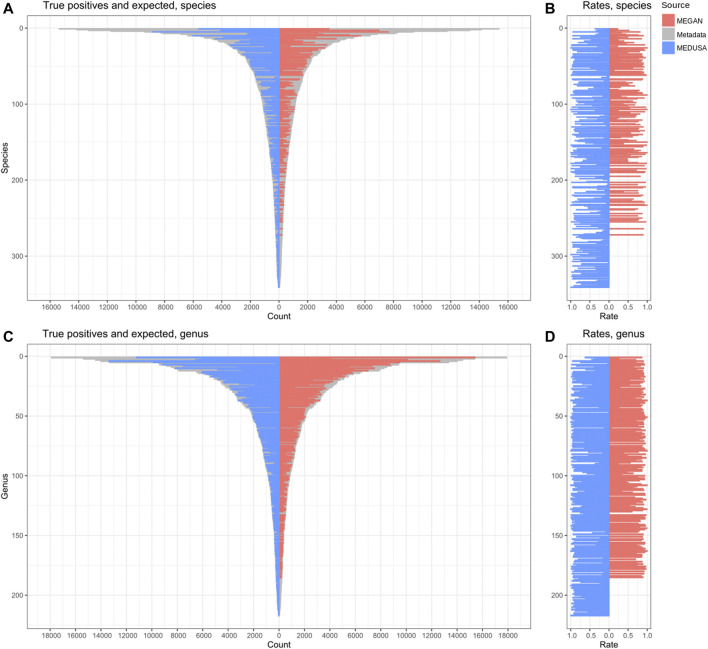
Reads correctly classified in the taxonomic analyses. True positives compared to the expected at species **(A)** and genus **(C)** levels. The proportion between these values at species **(B)** and genus levels **(D)**.

General information about the alignment and the analyses outputs are shown in Supplementary Tables S1–S9. The metrics resulting from the analyses of both simulated datasets are shown in Supplementary Tables S10, S11. To compute the MCC, allowing the functional result comparison, a true positive was defined as at least one expected GO ID assigned to a read. MEDUSA outperformed MEGAN in these functional results, with MEGAN obtaining a negative MCC (-0.22 MEGAN against 0.59 MEDUSA–Supplementary Table S11). Our pipeline assigned slightly more reads than MEGAN in the taxonomic analysis (Supplementary Table S2), and was much more efficient to identify the different species (51% MEGAN against 95% MEDUSA–Supplementary Table S12) and genus (78% MEGAN against 99% MEDUSA–Supplementary Table S12) present in D1 (Figure 6).

## 4 Discussion

Inspecting the results obtained by the tools benchmarks, and the comparison between MEGAN and MEDUSA, we outline the following findings. Fastp, an ultra-fast all-in-one FASTQ preprocessor, aggregates several useful features, being an excellent tool for preprocessing. As the DIAMOND aligner currently supports only SE reads, fastp is used after the host sequences removal to merge the PE reads. This contributes to minimizing the number of tools required to run the pipeline, avoiding a tool for the specific purpose of merging reads, such as PEAR (Zhang et al., 2014). Besides the implementation in C++, fastp runs faster by reading the FASTQ input only once. The report, saved in HTML and JSON, contains information about the reads before and after the processing. Bowtie2 obtained a low misclassification rate in our decontamination benchmark, and a recent study also chose Bowtie2 as the most suited tool to identify contaminants (Czajkowski et al., 2019). Kraken 2 might perform better using a different database, but we used the NCBI-nr as the reference protein database for all tools to conduct a fair comparison.

In D1 taxonomic results, the MEDUSA output was more standardized than the MEGAN output. Kaiju produces an output containing predetermined taxonomic ranks, defined by the user, being the following ranks used in our pipeline: superkingdom, phylum, class, order, family, genus, and species. The output from MEGAN contains descriptions like “NCBI” and “cellular organisms”, this counts as a valid classification but does not help to extract meaningful information from the results. The output from MEDUSA was more suited to estimate the correct taxonomic composition at species and genus levels. Furthermore, the less abundant species and genus were detected only by MEDUSA.

As the sequences used to create D2 are associated with 281 different GO terms, the criteria to count an annotation as a TP in the functional result is reasonably achievable. Yet, MEGAN obtained a negative MCC. In D2 functional results (Supplementary Tables S13–S18), MEDUSA assigned 303 distinct terms, while MEGAN assigned only 48. Besides, MEGAN assignments frequently include terms too broad, as the ontology roots shown in D3 functional results (Supplementary Tables S19–S23). This excessive presence of ontology roots hinders the extraction of biological insights. As D3 is a real dataset, we cannot measure the metrics without the ground truth, but both methodologies agree on the most abundant descriptions for the taxonomic results (Supplementary Tables S24–S26).

MEDUSA is a pipeline for shotgun metagenomic data deployed by the Conda package manager and managed by Snakemake. The Snakemake rules produce results for the reads with and without performing the assembly, but users can easily change this behavior by editing the rules. The intermediate files stored, that might be further inspected, are used by Snakemake to skip steps previously done when the pipeline is restarted. We also introduce annotate, an annotation transfer tool for user-built functional dictionaries. MEDUSA is easy to acquire, set up, and run, simplifying comprehensive metagenomic analyses. Advantages over MEGAN 6 CE involve more customizable thresholds to filter out alignment outputs for functional analysis, use of fast disk storage dictionaries created from plain text files and the flexibility to transfer any functional identifier, a more sensitive taxonomic classification, and fully automated steps to prepare the inputs for taxonomic and functional analyses.

As MEDUSA is obtained via the Conda package manager, additional software can be easily obtained using the Bioconda channel (Dale et al., 2018) to extend the pipeline. Similarly, one of the tools used by the pipeline can be replaced by one installed *via* Conda that produces a compatible output. To change the rules used during the pipeline execution, the user must edit the Snakefile, changing the commands called to run the software. New rules may be created in the Snakefile, written as Shell Script, and targets may be removed or included under the rule “all”. By default, our Snakefile has four targets that are related to taxonomic and functional outputs, half of them for analysis done with an assembly of contigs. If the user has no interest in an analysis performed with assembly, the two lines for the targets related to the contigs may be commented or deleted. This way, Snakemake will not perform any rule to create these files, as they are no more present in the targets.

## Data Availability

The original contributions presented in the study are included in the article/Supplementary Material and at https://github.com/dalmolingroup/MEDUSA_supplements, further inquiries can be directed to the corresponding author.

## Abbreviations

CAMI, critical assessment of metagenome interpretation; CE, community edition; D1, dataset 1; D2, dataset 2; D3, dataset 3; FN, false negatives; FP, false positives; GO, gene ontology; GRCh38, genome reference consortium human build 38; HTML, hypertext markup language; IDs, identifiers; JSON, Javascript object notation; MCC, Matthews correlation coefficient; NCBI, national center for biotechnology information; NCBI-nr, national center for biotechnology information—non-redundant; NC, negative control; PE, paired-end; SE, single-end; SRA, sequence read archive; TN, True Negatives; TP, true positives; UE, ultimate edition.
