# Supplementary material for: MEDUSA: A Pipeline for Sensitive Taxonomic Classification and Flexible Functional Annotation of Metagenomic Shotgun Sequences
Source: Front Genet. 2022 Mar 7;13:814437. doi: 10.3389/fgene.2022.814437 (PMC8940201; doi:10.3389/fgene.2022.814437)
Supplement: Supplementary file 1 [file DataSheet1.PDF]

Supplementary Material

Contents

Supplementary Tables S1-S26 .... **2**
Installing and running details **17**
1 Notation **17**
2 Materials **17**
3 Setup **17**
4 Folder structure **18**
5 Preprocessing **19**
6 Alignment **21**
7 Taxonomic classification **22**
8 Functional annotation **23**
9 Assembly **26**
10 Run with Snakemake **27**
Supplementary Notes #1-7 .... **29**
References **42**

Supplementary Table S1. General information about the simulated datasets.

	**Dataset 1**	**Dataset 2**
Number of reads generated	509688	500351
Number of reads after quality control	507429	498201
Number of reads aligned	389946	384952
Number of distinct bacterial sequences[(1)]	394	10
Number of distinct viral sequences[(1)]	40	0
Number of distinct archaeal sequences[(1)]	73	0
Number of distinct taxonomy identifiers[(2)]	407	10
Number of distinct seven-level taxonomic descriptions[(3)]	342	9
Number of negative control reads after quality control	99918	99918

[(1)]Downloaded by InSilicoSeq to generate the dataset

[(2)]Obtained by the NCBI's EDirect from the sequence identifiers

[(3)]Obtained by the ETE toolkit from the taxonomy identifiers

Supplementary Table S2. General information about the D1 taxonomic analyses outputs.

	**MEGAN**	**MEDUSA**
Total number of reads aligned	389946	-[(1)]
Assigned	385762 [(2)]	404854
Not assigned	4184	102575

[(1)]Kaiju does not require an alignment result as input

[(2)]Excluding only the entries for “NCBI;Not assigned;”, thus taken into account all other entries, such as the assigned to “NCBI;”, and “NCBI;cellular organisms;”

Supplementary Table S3. General information about the D1 taxonomic analyses outputs grouped by superkingdom.

	MEGAN	MEDUSA	Expected
Archaea	85713	89868	92845
Bacteria	249571	266856	264862
Eukaryota	256	0[1]	0
Viruses	45020	45969	49804

[1]Kaiju by default includes only Archaea, Bacteria, and Viruses sequences from NCBI-nr.

Supplementary Table S4. General information about the D2 functional analyses outputs.

	MEGAN	MEDUSA
Total number of reads aligned	384952	384952
Assigned	205463[1]	292847
Not assigned	179489	92105

[1]Taken into account only the reads assigned to at least one GO term

Supplementary Table S5. General information about the D2 functional analyses outputs grouped by ontology.

	MEGAN			MEDUSA		
	Biological Process	Cellular Component	Molecular Function	Biological Process	Cellular Component	Molecular Function
Number of entries[1]	19[2]	13[3]	16[4]	112	25	166

[1]Each entry is a distinct Gene Ontology term

[2]From which 6 are present in MEDUSA functional result

[3]From which 5 are present in MEDUSA functional result

[4]From which 6 are present in MEDUSA functional result

Supplementary Table S6. General information about the D3 taxonomic analyses outputs.

	MEGAN	MEDUSA
Total number of reads aligned	257567	-[(1)]
Assigned	251122 [(2)]	405860
Not assigned	6445	56157

[(1)] Kaiju does not require an alignment result as input

[(2)]Excluding only the entries for “NCBI;Not assigned;”, thus taken into account all other entries, such as the assigned to “NCBI;”, and “NCBI;cellular organisms;”

Supplementary Table S7. General information about the D3 taxonomic analyses outputs grouped by superkingdom.

	MEGAN	MEDUSA
Archaea	0	65
Bacteria	248786	395467
Eukaryota	0	0[(1)]
Viruses	0	4828

[(1)]Kaiju by default includes only Archaea, Bacteria, and Viruses sequences from NCBI-nr.

Supplementary Table S8. General information about the D3 functional analyses outputs.

	MEGAN	MEDUSA
Total number of reads aligned	257567	257567
Assigned	87935 [(1)]	150908
Not assigned	169632	106659

[(1)]Taken into account only the reads assigned to at least one GO term

Supplementary Table S9. General information about the D3 functional analyses outputs grouped by ontology.

	MEGAN			MEDUSA		
	**Biological Process**	**Cellular Component**	**Molecular Function**	**Biological Process**	**Cellular Component**	**Molecular Function**
Number of entries[(1)]	42[(2)]	29[(3)]	34[(4)]	1194	161	2013

[(1)]Each entry is a distinct Gene Ontology term

[(2)]From which 28 are present in MEDUSA functional result

[(3)]From which 21 are present in MEDUSA functional result

[(4)]From which 28 are present in MEDUSA functional result

Supplementary Table S10. Metrics related to the D1 taxonomic analyses outputs.

	MEGAN			MEDUSA		
	**Species**	**Genus**	**Phylum**	**Species**	**Genus**	**Phylum**
True Positive	181791	302092	326555	219421	301557	330160
True Negative	99918	99918	99918	95085	95085	95085
False Positive	203971	83670	59207	185433	103297	74694
False Negative	21749	21749	21749	7490	7490	7490
Sensitivity	0.8931	0.9328	0.9376	0.9670	0.9758	0.9778
Specificity	0.3288	0.5443	0.6279	0.3390	0.4793	0.5601
Positive Predictive Value	0.4713	0.7831	0.8465	0.5420	0.7449	0.8155
Negative Predictive Value	0.8212	0.8212	0.8212	0.9270	0.9270	0.927
Matthews Correlation Coefficient	0.2548	0.5370	0.6145	0.3788	0.5529	0.6319

Supplementary Table S11. Metrics related to the D2 functional analyses outputs.

	MEGAN	MEDUSA
True Positive[(1)]	88041	291641
True Negative	99918	99918
False Positive	117422	1206
False Negative	192820	105436
Sensitivity	0.3135	0.7345
Specificity	0.4597	0.9881
Positive Predictive Value	0.4285	0.9959
Negative Predictive Value	0.3413	0.4866
Matthews Correlation Coefficient	-0.2285	0.5904

[(1)]If at least one GO term assigned to the read matched any GO term related to its original sequence, this was counted as a true positive

Supplementary Table S12. Number of correctly assigned descriptions in the D1 taxonomic analyses outputs.

MEGAN			MEDUSA			Expected		
**Species**	**Genus**	**Phylum**	**Species**	**Genus**	**Phylum**	**Species**	**Genus**	**Phylum**
173	170	18	324	216	18	341	217	18

Supplementary Table S13. D2 summarised metadata.

GenBank	Number of distinct curated GO terms [1]	Number of reads generated
AB006738.1	13	104108
J02708.1	27	68124
D87215.1	13	65509
AF233324.1	193	48190
X56049.1	24	37290
X15079.1	11	31854
S81887.1	6	16013
L46864.1	10	10948
X05768.1	7	9063
U22037.1	28	7184

[1]The UniProt ID mapping API (https://www.uniprot.org/help/api_idmapping) was used to convert the GenBank IDs (EMBL_ID) to UniProt IDs (ACC), allowing to transfer the curated UniProtKB/Swiss-Prot Gene Ontology information from the UniProt IDs to the GenBank IDs

Supplementary Table S14. D2 MEGAN top 10 Gene Ontology terms found.

		MEGAN		MEDUSA	
**Description**	**Ontology**	**Position**	**Count**	**Position**	**Count**
Molecular function	MF	1	161749	-	-
Biological process	BP	2	159091	-	-
Cellular component	CC	3	144746	-	-
Catalytic activity	MF	4	139319	133	681
Metabolic process	BP	5	91652	272	21
Ion binding	MF	6	70641	-	-
Extracellular region	CC	7	46925	5	51537
Intrinsic component of membrane	CC	8	43154	-	-
Transport	BP	9	30143	-	-
Transporter activity	MF	10	24181	-	-

Supplementary Table S15. D2 MEDUSA top 10 Gene Ontology terms found.

		MEDUSA		MEGAN	
**Description**	**Ontology**	**Position**	**Count**	**Position**	**Count**
Integral component of membrane	CC	1	79075	-	-
Bacteriocin biosynthetic process	BP	2	65970	-	-
Plasma membrane	CC	3	59441	-	-
Pathogenesis	BP	4	52053	39	71
Extracellular region	CC	5	51537	7	46925
DNA binding	MF	6	45137	-	-
Zinc ion binding	MF	7	42298	-	-
Gamma-glutamyl carboxylase activity	MF	8	42105	-	-
Peptidyl-glutamic acid carboxylation	BP	9	42105	-	-
Cytoplasm	CC	10	42015	17	9244

Supplementary Table S16. D2 Top 10 Gene Ontology Biological processes found in the functional analyses outputs.

	MEGAN		MEDUSA	
**Position**	**Description**	**Count**	**Description**	**Count**
1	Biological process	159091	Bacteriocin biosynthetic process	65970
2	Metabolic process	91652	Pathogenesis	52053
3	Transport	30143	Peptidyl-glutamic acid carboxylation	42105
4	Bacterial-type flagellum-dependent cell motility	21172	Collagen metabolic process	41102
5	DNA metabolic process	12203	Phosphoenolpyruvate-dependent sugar phosphotransferase system	25860
6	Response to stress	8973	Bacterial-type flagellum assembly	19762
7	Cellular component organization or biogenesis	4424	Bacterial-type flagellum-dependent swarming motility	16325
8	Regulation of metabolic process	740	Bacterial-type flagellum-dependent cell motility	13522
9	RNA metabolic process	677	Cell killing	10435
10	Iron-sulfur cluster assembly	125	Cytolysis	10435

Supplementary Table S17. D2 Top 10 Gene Ontology Cellular components found in the functional analyses outputs.

	MEGAN		MEDUSA	
**Position**	**Description**	**Count**	**Description**	**Count**
1	Cellular component	144746	Integral component of membrane	79075
2	Extracellular region	46925	Plasma membrane	59441
3	Intrinsic component of membrane	43154	Extracellular region	51537
4	Membrane	9880	Cytoplasm	42015
5	Cytoplasm	9244	Bacterial-type flagellum hook	10987
6	Bacterial-type flagellum	6812	Type III protein secretion system complex	6611
7	Catalytic complex	780	Bacterial-type flagellum	6059
8	Membrane protein complex	687	Bacterial-type flagellum basal body	5652
9	Ribosome	80	Plasma membrane light-harvesting complex	5218
10	Periplasmic space	42	Cytosol	3779

Supplementary Table S18. D2 Top 10 Gene Ontology Molecular functions found in the functional analyses outputs.

	MEGAN		MEDUSA	
**Position**	**Description**	**Count**	**Description**	**Count**
1	Molecular function	161749	DNA binding	45137
2	Catalytic activity	139319	Zinc ion binding	42298
3	Ion binding	70641	Gamma-glutamyl carboxylase activity	42105
4	Transporter activity	24181	Calcium ion binding	41286
5	Nucleotide binding	12771	Endopeptidase activity	41102
6	Nucleic acid binding	11159	Metalloendopeptidase activity	41102
7	Tetrapyrrole binding	10749	Collagen binding	41102
8	Coenzyme binding	782	Tripeptidase activity	41102
9	Vitamin binding	641	ATP binding	22793
10	DNA-binding transcription factor activity	418	Protein-N(PI)-phosphohistidine-sugar phosphotransferase activity	20252

Supplementary Table S19. D3 MEGAN top 10 Gene Ontology terms found.

		MEGAN		MEDUSA	
**Description**	**Ontology**	**Position**	**Count**	**Position**	**Count**
Molecular function	MF	1	74324	-	-
Biological process	BP	2	69969	-	-
Catalytic activity	MF	3	51778	11	4099
Metabolic process	BP	4	41533	128	490
Cellular component	CC	5	29062	-	-
Nitrogen compound metabolic process	BP	6	17962	185	324
Small molecule metabolic process	BP	7	17838	-	-
Transferase activity	MF	8	16365	13	3958
Biosynthetic process	BP	9	15962	60	1145
Transport	BP	10	14707	-	-

Supplementary Table S20. D3 MEDUSA top 10 Gene Ontology terms found.

		MEDUSA		MEGAN	
**Description**	**Ontology**	**Position**	**Count**	**Position**	**Count**
Integral component of membrane	CC	1	41059	-	-
ATP binding	MF	2	26749	-	-
Cytoplasm	CC	3	18621	28	2800
DNA binding	MF	4	17461	-	-
Metal ion binding	MF	5	11218	22	4474
Plasma membrane	CC	6	10229	60	438
Hydrolase activity	MF	7	5990	12	13510
ATPase activity	MF	8	5961	-	-
Carbohydrate metabolic process	BP	9	5956	19	6810
Zinc ion binding	MF	10	4132	-	-

Supplementary Table S21. D3 Top 10 Gene Ontology Biological processes found in the functional analyses outputs.

	MEGAN		MEDUSA	
**Position**	**Description**	**Count**	**Description**	**Count**
1	Biological process	69969	Carbohydrate metabolic process	5956
2	Metabolic process	41533	Transmembrane transport	3928
3	Nitrogen compound metabolic process	17962	Regulation of transcription, DNA-templated	3167
4	Small molecule metabolic process	17838	DNA recombination	2214
5	Biosynthetic process	15962	DNA replication	2056
6	Transport	14707	Methylation	1905
7	Carbohydrate metabolic process	6810	DNA integration	1861
8	RNA metabolic process	5653	Transposition, DNA-mediated	1833
9	DNA metabolic process	4352	Cell division	1829
10	Generation of precursor metabolites and energy	2917	DNA repair	1789

Supplementary Table S22. D3 Top 10 Gene Ontology Cellular components found in the functional analyses outputs.

	MEGAN		MEDUSA	
**Position**	**Description**	**Count**	**Description**	**Count**
1	Cellular component	29062	Integral component of membrane	41059
2	Membrane	9900	Cytoplasm	18621
3	Intrinsic component of membrane	8631	Plasma membrane	10229
4	Cytoplasm	2800	Membrane	1359
5	Ribosome	2543	Integral component of plasma membrane	1331
6	Catalytic complex	1717	Ribosome	1257
7	Membrane protein complex	1523	ATP-binding cassette (ABC) transporter complex	1164
8	Oxidoreductase complex	482	Cell outer membrane	1097
9	ATP-binding cassette (ABC) transporter complex	455	Cytosol	1085
10	Plasma membrane	438	Cell	772

Supplementary Table S23. D3 Top 10 Gene Ontology Molecular functions found in the functional analyses outputs.

	MEGAN		MEDUSA	
**Position**	**Description**	**Count**	**Description**	**Count**
1	Molecular function	74324	ATP binding	26749
2	Catalytic activity	51778	DNA binding	17461
3	Transferase activity	16365	Metal ion binding	11218
4	Ion binding	14414	Hydrolase activity	5990
5	Hydrolase activity	13510	ATPase activity	5961
6	Transporter activity	11132	Zinc ion binding	4132
7	Nucleotide binding	10736	Catalytic activity	4099
8	Oxidoreductase activity	9009	Magnesium ion binding	3982
9	Nucleic acid binding	7934	Transferase activity	3958
10	Ligase activity	4740	DNA-binding transcription factor activity	3898

Supplementary Table S24. D3 MEGAN top 10 taxonomic descriptions found in the Bacteria superkingdom.

		MEGAN		MEDUSA	
**Description**	**Rank**	**Position**	**Count**	**Position**	**Count**
Clostridiales	Order	1	43949	-	-
Firmicutes	Phylum	2	20213	3	40881
Akkermansia muciniphila	Species	3	19338	6	19579
Akkermansia	Genus	4	17644	4	29352
Enterobacteriaceae	Family	5	17313	7	13331
Lachnospiraceae	Family	6	13734	5	22509
Bacteria	Superkingdom	7	10902	2	43121
Escherichia coli	Species	8	8357	8	12624
Blautia sp. CAG:257	Species	9	7965	31	1193
Streptococcus	Family	10	6007	9	8981

Supplementary Table S25. D3 MEDUSA top 10 taxonomic descriptions found in the Bacteria superkingdom.

		MEDUSA		MEGAN	
**Description**	**Rank**	**Position**	**Count**	**Position**	**Count**
Eubacteriales	Order	1	91441	-	-
Bacteria	Superkingdom	2	43121	7	10902
Firmicutes	Phylum	3	40881	2	20213
Akkermansia	Genus	4	29352	4	17644
Lachnospiraceae	Family	5	22509	6	13734
Akkermansia muciniphila	Species	6	19579	3	19338
Enterobacteriaceae	Family	7	13331	5	17313
Escherichia coli	Species	8	12624	8	8357
Streptococcus	Family	9	8981	10	6007
Clostridium	Genus	10	8773	32	1024

Supplementary Table S26. D3 number of entries and count sum from the top descriptions found in the Bacteria superkingdom of both methodologies.

	MEGAN		MEDUSA	
**Description**	**Number of entries**	**Count sum**	**Number of entries**	**Count sum**
Clostridiales	99	108855	-	-
Firmicutes	138	152915	2277	244897
Akkermansia muciniphila	1	19338	1	19579
Akkermansia	5	38419	14	49568
Enterobacteriaceae	10	28659	118	29011
Lachnospiraceae	54	43218	421	52889
Bacteria	194	248786	4006	395467
Escherichia coli	1	8357	1	12624
Blautia sp. CAG:257	1	7965	1	1193
Streptococcus	3	6631	69	10760
Eubacteriales	-	-	1307	172567
Clostridium	8	8922	249	13878

Installing and running details

1 Notation

In what follows, all commands run at the command line and R scripts appear in **`bold Courier`** font. Unix commands are prefaced by a dollar sign ($):

```
$ wc -l file.txt
```

R scripts are prefaced by a greater-than sign (>):

```
> mean(1:10)
```

Both left aligned.

2 Materials

2.1 Operating system

This pipeline assumes that users have a Linux operating system with a bash shell or similar. All commands given here were tested on Ubuntu 18.04, and are meant to be run in a terminal window.

2.2 Input file

The initial input is meant to be a FASTQ file, a common format for reads from Illumina sequencing machines.

2.3 Example data

We selected a public human gut metagenome shotgun data from the National Center for Biotechnology Information (NCBI). The run SRR579292 from the BioProject PRJNA175224 is a paired-end data obtained by an Illumina MiSeq instrument, a sequencing platform which produces a maximum output of a few GB. The chosen run is a small dataset from the NCBI Sequence Read Archive (SRA), being suited for a quick demonstration of our pipeline. This data is from a patient with Crohn's disease and the following analysis aims to identify the microbial community and the functional activity present within it.

3 Setup

An Anaconda environment was created to ease the installation of all required software and is available at the anaconda cloud (https://anaconda.org/arthurvinx/medusapipeline). A conda

package manager, such as miniconda (https://docs.conda.io/en/latest/miniconda.html), must be installed to get this environment.

Download and install the latest miniconda release for Python 3.7 (adapt the commands if needed):
```
$ cd ~/Downloads
$ chmod u+x Miniconda3-latest-Linux-x86_64.sh
$ ./Miniconda3-latest-Linux-x86_64.sh
```

Check if the installation was successful (need to exit the terminal and then return to it):
```
$ conda -V
```

Get the environment containing the required tools to run the protocol:
```
$ conda activate base
$ conda install anaconda-client -y
$ conda env create arthurvinx/medusaPipeline
$ conda activate medusaPipeline
$ pip3 install -U plyvel --no-cache-dir --no-deps --force-
reinstall
```

This environment must be active to run the commands from the next sections.

Activate the environment:
```
$ conda activate medusaPipeline
```

4 Folder structure

This pipeline assumes that the following folder structure will be used to organise the downloaded data, intermediate files, and outputs.

```
.
└── Pipeline
    ├── alignment
    │   ├── db
    │   └── index
    ├── data
    │   ├── assembled
    │   ├── collapsed
    │   ├── merged
    │   ├── raw
    │   ├── removal
    │   │   ├── index
```

```
│   │   └── reference
│   └── trimmed
├── functional
│   └── db
├── result
└── taxonomic
    └── db
```

Create this folder structure:

```
$ mkdir -p
./Pipeline/{result,data/{merged,assembled,collapsed,removal/{ind
ex,reference},raw,trimmed},alignment/{db,index},taxonomic/db,fun
ctional/db}
```

5 Preprocessing

Change the current directory to “Protocol/data” and download the example data:

```
$ prefetch SRR579292 -X 20G
```

Note: In the prefetch call, the -X argument specifies the maximum file size to download (adapt this argument if needed).

Get the paired-end reads:

```
$ fasterq-dump SRR579292 -e 8
```

Note: In the fasterq-dump call, the -e argument specifies the number of threads to use (adapt this argument if needed).

Remove low-quality sequences and adapters:

```
$ fastp -i SRR579292_1.fastq -I SRR579292_2.fastq -o
trimmed/SRR579292_1_trim.fastq -O trimmed/SRR579292_2_trim.fastq
-q 20 -w 8 --detect_adapter_for_pe -h trimmed/report.html -j
trimmed/fastp.json
```

Note: In the fastp call, the -q argument sets the phred quality score threshold, and -w specifies the number of threads to use (adapt these arguments if needed).

A *Homo sapiens* reference genome is needed to remove the host sequences from this data. Change the current directory to “Pipeline/data/removal/reference” and download a reference genome from Ensembl:

```
$ wget ftp://ftp.ensembl.org/pub/release-
102/fasta/homo_sapiens/dna/Homo_sapiens.GRCh38.dna.primary_assem
bly.fa.gz
$ pigz -d Homo_sapiens.GRCh38.dna.primary_assembly.fa.gz -p 8
```

Note: The pigz software is a parallel implementation of gzip, the -p argument specifies the number of threads to use.

Build a bowtie2 index:

```
$ bowtie2-build Homo_sapiens.GRCh38.dna.primary_assembly.fa
../index/host --threads 8
```

Go back to “Pipeline/data” and align the sequences against the reference:

```
$ bowtie2 -x removal/index/host -1
trimmed/SRR579292_1_trim.fastq -2 trimmed/SRR579292_2_trim.fastq
-S removal/all.sam -p 8
```

Extract the unaligned reads:

```
$ samtools view -bS removal/all.sam > removal/all.bam
$ samtools view -b -f 12 -F 256 removal/all.bam >
removal/unaligned.bam
$ samtools sort -n removal/unaligned.bam -o
removal/unaligned_sorted.bam
$ samtools bam2fq removal/unaligned_sorted.bam >
removal/unaligned.fastq
$ cat removal/unaligned.fastq | grep '^@.*/1$' -A 3 --no-group-
separator > removal/unaligned_1.fastq
$ cat removal/unaligned.fastq | grep '^@.*/2$' -A 3 --no-group-
separator > removal/unaligned_2.fastq
```

Note: In the SAMtools call, the -f and -F arguments use flags to specify, respectively, which alignments must be extracted and which ones should not be extracted. See http://broadinstitute.github.io/picard/explain-flags.html to know more about the available SAMtools flags.

Merge the paired-end reads:

```
$ fastp -i removal/unaligned_1.fastq -I
removal/unaligned_2.fastq -o trimmed/unmerged_1.fastq -O
trimmed/unmerged_2.fastq -q 20 -w 8 --detect_adapter_for_pe -h
trimmed/report2.html -j trimmed/fastp2.json -m --merged_out
merged/SRR579292_merged.fastq
```

Remove duplicated sequences:

```
$ fastx_collapser -i merged/SRR579292_merged.fastq -o
collapsed/SRR579292.fasta
```

6 Alignment

Change the current directory to "Pipeline/alignment/db" and download the NCBI-nr protein database:

```
$ wget ftp://ftp.ncbi.nlm.nih.gov/blast/db/FASTA/nr.gz
$ pigz -d nr.gz -p 8
```

Build a DIAMOND index:

```
$ diamond makedb --in nr -d ../index/nr
```

Change the current directory to "Pipeline/alignment" and align the reads against the reference protein database:

```
$ touch unaligned.fasta
$ diamond blastx -d index/nr -q
../data/collapsed/SRR579292.fasta -o matches.m8 --top 3 --un
unaligned.fasta
```

Note: In the DIAMOND call, the --un argument specifies the file used to write the unaligned sequences, and --top 3 report alignments within this percentage range of top alignment score. DIAMOND may use a lot of memory and temporary disk space. Therefore, the program may fail due to running out of either one. The -b argument specifies the block size argument, and -c the number of chunks for index processing. The total memory usage can be roughly estimated by 2(b+9b/c) GB. On a high memory server, set -c to 1.

Perform a more sensitive alignment using the unaligned sequences:

```
$ touch unaligned2.fasta
$ diamond blastx -d index/nr -q unaligned.fasta -o matches2.m8 -
-more-sentitive --top 3 --un unaligned2.fasta
```

Note: The default sensitive mode is designed for short reads (around 100 bp), finding hits of >60% identity. For longer sequences, the sensitive modes are recommended. DIAMOND is much more efficient for large query files (> 1 million reads).

Check the number of queries presenting hits with at least 80% of identity in matches2.m8:

```
$ awk '{ if ($3>=80) { print } }' matches2.m8 > check.m8
```

```
$ awk '{a[$1]++}END{for (i in a) sum+=1; print sum}' check.m8
```

Concatenate the alignment outputs:

```
$ cat matches.m8 matches2.m8 > all_matches.m8
```

7 Taxonomic classification

Change the current directory to “Pipeline/taxonomic/db” and download the following files:

```
$ wget ftp://ftp.ncbi.nlm.nih.gov/pub/taxonomy/taxdump.tar.gz
$ wget
ftp://ftp.ncbi.nlm.nih.gov/pub/taxonomy/accession2taxid/prot.acc
ession2taxid.gz
```

Extract and decompress the required files:

```
$ tar -xf taxdump.tar.gz nodes.dmp names.dmp
$ pigz -d prot.accession2taxid.gz -p 8
```

Build a Kaiju index:

```
$ kaiju-convertNR -t nodes.dmp -g prot.accession2taxid -e
~/miniconda3/envs/medusaPipeline/bin/kaiju-excluded-
accessions.txt -a -o kaijuNR.fasta -i ../../alignment/db/nr
$ kaiju-mkbwt -n 8 -a ACDEFGHIKLMNPQRSTVWY -o kaijuNR
kaijuNR.fasta
$ kaiju-mkfmi kaijuNR
```

Note: In the kaiju-convertNR call, by default, are included only Archaea, Bacteria, and Viruses sequences from the NCBI-nr. This behavior can be changed by the -l argument, passing an input file as ~/miniconda3/envs/medusaPipeline/bin/kaiju-taxonlistEuk.tsv. This argument uses only sequences with ancestors listed in the file.

Change the current directory to “Pipeline/taxonomic” and perform the taxonomic classification:

```
$ kaiju -t db/nodes.dmp -f db/kaijuNR.fmi -i
../data/removal/unaligned_1.fastq -j
../data/removal/unaligned_2.fastq -o
../result/SRR579292_kaiju.out -z 8
```

Add taxa names to the output:

```
$ kaiju-addTaxonNames -t db/nodes.dmp -n db/names.dmp -r
superkingdom,phylum,class,order,family,genus,species -i
../result/SRR579292_kaiju.out -o ../result/SRR579292_kaiju.names
```

Generate the Krona plots:
```
$ kaiju2krona -t db/nodes.dmp -n db/names.dmp -i
../result/SRR579292_kaiju.out -o
../result/SRR579292_kaiju2krona.out
$ ktImportText -o ../result/SRR579292_krona.html
../result/SRR579292_kaiju2krona.out
```

8 Functional annotation

Change the current directory to "Pipeline/functional" and download the UniProt ID mapping file:
```
$ wget
ftp://ftp.uniprot.org/pub/databases/uniprot/current_release/know
ledgebase/idmapping/idmapping_selected.tab.gz
$ pigz -d idmapping_selected.tab.gz -p 8
```

Filter the columns containing Gene Ontology and GenBank identifiers:
```
$ awk -F "\t" '{if(($7!="") && ($18!="")){print $18"\t"$7}}'
idmapping_selected.tab > genbank2GO.txt
```

Filter the columns containing Gene Ontology and RefSeq identifiers:
```
$ awk -F "\t" '{if(($4!="") && ($7!="")){print $4"\t"$7}}'
idmapping_selected.tab > refseq2GO.txt
```

Use the R language to clean the dictionaries:
```
> library(dplyr)
> library(tidyr)
> library(data.table)
> fixCollapsed <- function(df){
  colnames(df) <- c("key", "value")
  df <- df %>%
     mutate(key = strsplit(key, "; ")) %>%
     unnest(key)
  df <- df[, c(2, 1)]
  return(df)
}
> fixDuplicated <- function(df){
  df <- df %>%
     group_by(key) %>%
     summarise(value = paste(value, collapse = "; "))
```

```
  values <- strsplit(df$value, "; ")
  values <- lapply(values, unique)
  values <- sapply(values, paste, collapse = "; ")
  df$value <- values
  return(df)
}
> removeUnknown <- function(df){
  idx <- grepl("^-", df$key)
  df <- df[!idx,]
  return(df)
}
> df <- fread("genbank2GO.txt", stringsAsFactors = FALSE,
head = FALSE, nThread = parallel::detectCores())
> df <- as.data.frame(df)
> df %>%
  fixCollapsed() %>%
  fixDuplicated() %>%
  removeUnknown() %>%
  fwrite(file = "NR2GO.txt", sep = "\t",
nThread = parallel::detectCores())
> df <- fread("refseq2GO.txt", stringsAsFactors = FALSE,
head = FALSE, nThread = parallel::detectCores())
> df <- as.data.frame(df)
> df %>%
  fixCollapsed() %>%
  fixDuplicated() %>%
  removeUnknown() %>%
  fwrite(file = "NR2GO.txt", sep = "\t",
append = TRUE, nThread = parallel::detectCores())
```

Note: This script requires a lot of memory to deal with the filtered UniProt ID mapping file contents. A 4.9 GB input requires roughly 78 GB of memory.

Generate a levelDB from the cleaned dictionary using annotate:

```
$ annotate createdb NR2GO.txt NR2GO 0 1 -d db
```

Note: More information about annotate can be found at https://github.com/dalmolingroup/annotate.

Annotate each query using the best alignment for which a mapping is known:

```
$ annotate idmapping ../alignment/all_matches.m8
../result/SRR579292_functional_GO.txt NR2GO -l 1 -d db
```

Note: In the annotate call, the -l argument modifies the alignment length threshold.

Use the R language to get the GO identifiers description:

```
> library(GO.db)
> library(data.table)
> library(dplyr)
> library(tidyr)
> fixCollapsed <- function(df){
  df <- df %>%
     mutate(Annotation = strsplit(Annotation, "; ")) %>%
     unnest(Annotation)
  df <- df[, c(2, 1)]
  return(df)
}
> getGODescription <- function(GOs){
  BP <- names(as.list(GO.db::GOBPCHILDREN))
  CC <- names(as.list(GO.db::GOCCCHILDREN))
  MF <- names(as.list(GO.db::GOMFCHILDREN))
  result <- vapply(GOs, function(x){
     if(x %in% BP){
     return("BP")
     }else if(x %in% CC){
     return("CC")
     }else if(x %in% MF){
     return("MF")
     }else{
     return(NA_character_)
     }
  }, character(1))
  return(result)
}
> data <- as.data.frame(fread("functional.txt", header = T,
stringsAsFactors = F))
> data <- fixCollapsed(data)
> data <- data$Annotation
> data <- as.data.frame(table(data))
> data$data <- as.character(data$data)
```

```
> data$Ontology <- getGODescription(data$data)
> colnames(data)[1] <- "GOId"
> idx <- which(is.na(data$Ontology))
> data$Ontology[idx] <- "Unknown"
> xx <- as.list(GOTERM)
> data$desc <- sapply(data$GOId, function(i){
  flagError <- FALSE
  tryCatch({
     res <- xx[[i]]@Term
  }, error = function(e) {
     flagError <<- TRUE
  })
  if(flagError){
     return("Unknown")
  }
  else{
     return(res)
  }
})
> data <- data[, c(2, 3, 1, 4)]
> write.table(data, file = "functional_description.txt",
col.names = TRUE, row.names = FALSE, quote = FALSE, sep = "\t")
```

9 Assembly

Change the current directory to “Pipeline/data/assembled” and assemble the decontaminated reads into contigs using MEGAHIT:

```
$ megahit -1 ../removal/unaligned_1.fastq -2
../removal/unaligned_2.fastq -o SRR579292 -t 8
```

Use Kaiju to classify the contigs:

```
$ kaiju -t ../../taxonomic/db/nodes.dmp -f
../../taxonomic/db/kaijuNR.fmi -i SRR579292/final.contigs.fa -o
../../result/SRR579292_contigs_kaiju.out -z 8
```

The Kaiju call in section 7 used a paired-end input, while this call use the contigs as a single-end input. The other commands previously used to perform the taxonomic classification and the functional annotation, in sections 7 and 8 respectively, may be used to perform these analyses on this Kaiju output and the contigs.

10 Run with Snakemake

10.1 Download the GitHub repository

Download the GitHub repository (https://github.com/dalmolingroup/MEDUSA) to get the files describing the pipeline rules:

```
$ wget
https://github.com/dalmolingroup/MEDUSA/archive/refs/heads/main.
zip
$ unzip main.zip
$ cd Medusa-main/
```

10.2 Create the expected folder structure

Go to the folder containing the Snakefile (via command line) and create the expected folder structure with:

```
$ mkdir -p
./Pipeline/{result,data/{merged,assembled,collapsed,removal/{ind
ex,reference},raw,trimmed},alignment/{db,index},taxonomic/db,fun
ctional/db}
```

10.3 Install the conda package manager

An Anaconda environment was created to ease the installation of all required software and is available at the anaconda cloud (https://anaconda.org/arthurvinx/medusapipeline). A conda package manager, such as miniconda (https://docs.conda.io/en/latest/miniconda.html), must be installed to get this environment.

Download and install the latest miniconda release for Python 3.7 (adapt the commands if needed):

```
$ cd ~/Downloads
$ chmod u+x Miniconda3-latest-Linux-x86_64.sh
$ ./Miniconda3-latest-Linux-x86_64.sh
```

Check if the installation was successful (need to exit the terminal and then return to it):

```
$ conda -V
```

10.4 Get the pipeline environment

Get the environment containing the required tools to run the protocol:

```
$ conda activate base
```

```
$ conda install anaconda-client -y
$ conda env create arthurvinx/medusaPipeline
$ conda activate medusaPipeline
$ pip3 install -U plyvel --no-cache-dir --no-deps --force-
reinstall
```

10.5 Install Snakemake

The recommended way to install Snakemake is via the conda package manager. The following commands will create a conda environment with the full version of Snakemake. More details can be found at the Snakemake installation guide (https://snakemake.readthedocs.io/en/stable/getting_started/installation.html).

```
$ conda activate base
$ conda install -c conda-forge mamba -y
$ mamba create -c bioconda -c conda-forge -n snakemake snakemake
```

Check whether your installation succeeded by typing:

```
$ conda activate snakemake
$ snakemake –help
```

10.6 Move the input files to the expected location

Move your raw FASTQ files to the inputDIR specified in the Snakefile. By default, the inputDIR is "Pipeline/data/raw" and paired-end filenames are expected to present the suffixes "_1.fastq" and "_2.fastq". Alternatively, you may change the inputDIR editing the Snakefile. It is worth to mention that all paths in the Snakefile are interpreted relative to the directory Snakemake is executed in.

10.7 Run Snakemake

To start this pipeline, go to the folder containing the Snakefile (via command line) and run:

```
$ snakemake --cores
```

This will use all available cores whenever possible. Alternatively, you may define the number of available cores as seen in the Snakemake command line interface guide (https://snakemake.readthedocs.io/en/stable/executing/cli.html).

Supplementary Note #1: Creating the simulated datasets

A positive control was created using the InSilicoSeq software Illumina MiSeq model (https://insilicoseq.readthedocs.io/). For the D1, sequences were randomly downloaded from NCBI using:

```
$ iss generate --cpus 8 --model miseq -k bacteria -U 400 --
n_reads 800 --output sim1BAC --quiet
$ iss generate --cpus 8 --model miseq -k archaea -U 80 --n_reads
800 --output sim1ARC --quiet
$ iss generate --cpus 8 --model miseq -k viruses -U 40 --n_reads
800 --output sim1VIR --quiet
```

Duplicated sequences were removed, resulting in 394 bacterial, 73 archaeal, and 40 viral sequences. Reads were generated in a log-normal distribution for each file separately, using the --genomes argument from InSilicoSeq. Around 250, 100, and 50 thousand reads were created from the bacterial, archaeal, and viral sequences, respectively.

For the negative control, 200 bacterial sequences were randomly downloaded. After removing duplicates, 199 sequences remained and were shuffled using the esl-shuffle program from HMMER 3.3 (http://hmmer.org/):

```
$ esl-shuffle -o NC_shuffled.fasta -w 500 NC_Pool.fasta
```

Using these shuffled sequences as input for the InSilicoSeq software, around 100,000 reads were generated.

For the D2, the following sequences were selected:

AB006738.1 (https://www.ncbi.nlm.nih.gov/nuccore/AB006738.1/) - Relates to the sdpA-sdpB-sdpC operon in *Bacillus subtilis*, a sporulation delay system that acts via cannibalism, *i.e.*, the production of toxins to induce cell killing/cytolysis in neighboring genetically identical cells.

J02708.1 (https://www.ncbi.nlm.nih.gov/nuccore/J02708.1) - Refers to PTS, a carbohydrate active transport system in *Escherichia coli*. This sequence also includes genes related to peptidoglycan cleavage, playing a role in cell division and cell wall biogenesis.

D87215.1 (https://www.ncbi.nlm.nih.gov/nuccore/D87215.1) - Encodes the collagenase colG. Containing a catalytic zinc site and calcium-dependent domains, for the enzymatic and collagen binding activities, respectively. Acting as a gateway for carbon consumption in saprophytes such as *Hathewaya histolytica*.
AF233324.1 (https://www.ncbi.nlm.nih.gov/nuccore/AF233324.1) - Refers to a large (around 96,000 base-pairs) segment from the *Salmonella typhimurium* genome. This sequence includes genes ranging from cAMP biosynthesis (cyaA), amino acid (rhtC) and ion transport (corA), and membrane biogenesis (wecA).

X56049.1 (https://www.ncbi.nlm.nih.gov/nuccore/X56049.1) - Refers to a very expansive system of flagellum assembly and regulation, including chemotaxis and energy transduction. This sequence is from *Bacillus subtillis*, known for its high motility.

X15079.1 (https://www.ncbi.nlm.nih.gov/nuccore/X15079.1) - Refers to a phosphorylation-associated regulatory system (fixJ-fixK), responsible for the transcriptional activation, or repression, of nitrogen fixation genes. This sequence is from *Rhizobium meliloti*, known for its role in the nitrogen cycle.

S81887.1 (https://www.ncbi.nlm.nih.gov/nuccore/S81887.1) - Encodes an iron-sulphur flavoprotein with a methane monooxygenase activity in the methanotroph *Methylosinus trichosporium*, being responsible for the initial conversion of methane to methanol in this bacteria's metabolism.

L46864.1 (https://www.ncbi.nlm.nih.gov/nuccore/L46864.1) - Corresponds to the gene cadA, responsible for an ATPase ion transport pump of cadmium, zinc, and cobalt. Therefore, correlated with functions such as ATP binding, zinc ion transport, and hydrolase activity. The efflux of these ions to *Helicobacter pylori* directly influences the activity of the urease enzyme, which is necessary for its survival in the acidic gastric environment.

X05768.1 (https://www.ncbi.nlm.nih.gov/nuccore/X05768.1) - Encodes a tetraheme cytochrome molecule present in the photosynthetic reaction center of purple bacteria, such as *Blastochloris viridis*, being responsible for the reduction of the primary electron donor.

U22037.1 (https://www.ncbi.nlm.nih.gov/nuccore/U22037.1) - Refers to the two-component regulatory system DevR/DevS, associated with the hypoxic response in *Mycobacterium tuberculosis*, encoding sensor kinases that contribute to dormancy.

Using these sequences, around 400,000 reads were generated by the InSilicoSeq software. Finally, the negative control reads were integrated to both datasets.

Supplementary Note #2: Annotate

Annotate is a Python 3 script that annotates each query, from a BLAST/DIAMOND tabular output, using the best alignment for which a mapping is known. The MEDUSA pipeline uses this script to perform the functional annotation, filtering the alignments according to user-defined thresholds, and assigning functional identifiers to the best alignment possible of each read. Alignments not meeting thresholds for e-value, bit-score, percent identity, or alignment length, are ignored. If a read contains no alignment passing the thresholds, or none could be mapped, it is assigned to "Unknown". The memory required to generate the output is minimal as the input is processed linearly. Furthermore, it is also possible to omit unknown mappings from the output or to map all the alignments. Annotate uses LevelDB, a fast key-value disk storage developed by Google, to perform random searches. LevelDB random read performance can be seen at https://github.com/google/leveldb#read-performance. Some tools allow the creation of custom databases to perform the alignment, but we emphasize that annotate works differently. Annotate works with the alignment results, avoiding the need to perform a new alignment to obtain different functional identifiers. Users can build dictionaries from plain text files to map the alignment results to KEGG (Kyoto Encyclopedia of Genes and Genomes) Enzyme Commission numbers, Gene Ontology terms, InterPro, Entrez, UniProt, or any other identifier. The biomaRt R/Bioconductor package, and the UniProt Retrieve/ID mapping tool may help to build the dictionaries. Besides, users can easily filter alignments with annotate's arguments. Annotate's documentation, and an example of usage, can be seen at https://github.com/dalmolingroup/annotate.

Supplementary Note #3: Taxonomic and functional comparisons results

The raw sequences in FASTQ format, simulated datasets metadata, analyses results, and scripts used to compare MEDUSA and MEGAN, are available at https://github.com/dalmolingroup/ComparisonMEGANxMEDUSA.

Supplementary Note #4: Hardware used in the benchmarks

All benchmarks were run in the High-Performance Computing Center (NPAD) at the Federal University of Rio Grande do Norte (UFRN). Computational nodes with two 16-core Intel Xeon E5-2698v3 processors and 128 GB DDR4 RAM were requested exclusively for each benchmark.

Supplementary Note #5: Tools versions

All the tools and dependencies used, and their respective versions, are listed in the conda recipe that can be exported from the conda environment created, or downloaded at https://anaconda.org/arthurvinx/medusaPipeline/2021.05.19.1107/download/medusaPipeline.yaml.

Supplementary Note #6: Matthews Correlation Coefficient

The Matthews Correlation Coefficient (MCC) is a metric used to evaluate the confusion matrix in binary classification problems. The MCC ranges from -1 to +1, producing a score that reflects the ratio between positive and negative elements, even for imbalanced datasets (Chicco & Jurman, 2020). The decontamination benchmark is a binary classification problem, in which we assess the classification quality with human and non-human reads. In the taxonomic and functional benchmarks, we aggregated each one of the four confusion matrix categories, using the micro-average to assess the classification quality.

Supplementary Note #7: Benchmarks

The run SRR5371509, from the Sequence Read Archive (SRA), was used to benchmark the trimming tools. The reads were split in into files containing 1, 5, 10, and 40 million reads:

```
$ head -n 4000000 SRR5371509.sra_1.fastq > R1_1M.fastq
$ head -n 20000000 SRR5371509.sra_1.fastq > R1_5M.fastq
$ head -n 40000000 SRR5371509.sra_1.fastq > R1_10M.fastq
$ head -n 160000000 SRR5371509.sra_1.fastq > R1_40M.fastq
$ head -n 4000000 SRR5371509.sra_2.fastq > R2_1M.fastq
$ head -n 20000000 SRR5371509.sra_2.fastq > R2_5M.fastq
$ head -n 40000000 SRR5371509.sra_2.fastq > R2_10M.fastq
$ head -n 160000000 SRR5371509.sra_2.fastq > R2_40M.fastq
```

And then processed by the tools:

```
$ echo FASTP
$ /usr/bin/time -f "%E time_elapsed\n%S time_sys\n%U
time_user\n%P perc_cpu\n%M mem_max(Kb)" fastp -i R1_5M.fastq -o
SE1_trim.fastq -q 20 -w 1 -h fastpSE1.html -j fastpSE1.json --
adapter_sequence=AGATCGGAAGAGCACACGTCTGAACTCCAGTCA
2>fastpSE1.txt
$ /usr/bin/time -f "%E time_elapsed\n%S time_sys\n%U
time_user\n%P perc_cpu\n%M mem_max(Kb)" fastp -i R1_5M.fastq -o
```

```
SE2_trim.fastq -q 20 -w 4 -h fastpSE2.html -j fastpSE2.json --
adapter_sequence=AGATCGGAAGAGCACACGTCTGAACTCCAGTCA
2>fastpSE2.txt
$ /usr/bin/time -f "%E time_elapsed\n%S time_sys\n%U
time_user\n%P perc_cpu\n%M mem_max(Kb)" fastp -i R1_5M.fastq -I
R2_5M.fastq -o PE1_1_trim.fastq -O PE1_2_trim.fastq -q 20 -w 1 -
h fastpPE1.html -j fastpPE1.json --
adapter_sequence=AGATCGGAAGAGCACACGTCTGAACTCCAGTCA --
adapter_sequence_r2=AGATCGGAAGAGCGTCGTGTAGGGAAAGAGTGT --
detect_adapter_for_pe 2>fastpPE1.txt
$ /usr/bin/time -f "%E time_elapsed\n%S time_sys\n%U
time_user\n%P perc_cpu\n%M mem_max(Kb)" fastp -i R1_5M.fastq -I
R2_5M.fastq -o PE2_1_trim.fastq -O PE2_2_trim.fastq -q 20 -w 4 -
h fastpPE2.html -j fastpPE2.json --
adapter_sequence=AGATCGGAAGAGCACACGTCTGAACTCCAGTCA --
adapter_sequence_r2=AGATCGGAAGAGCGTCGTGTAGGGAAAGAGTGT --
detect_adapter_for_pe 2>fastpPE2.txt
$ echo AFTERQC
$ /usr/bin/time -f "%E time_elapsed\n%S time_sys\n%U
time_user\n%P perc_cpu\n%M mem_max(Kb)" after.py -1 R1_5M.fastq
-q 20 2>afterqcSE1.txt
$ /usr/bin/time -f "%E time_elapsed\n%S time_sys\n%U
time_user\n%P perc_cpu\n%M mem_max(Kb)" after.py -1 R1_5M.fastq
-2 R2_5M.fastq -q 20 2>afterqcPE1.txt
$ echo CUTADAPT
$ /usr/bin/time -f "%E time_elapsed\n%S time_sys\n%U
time_user\n%P perc_cpu\n%M mem_max(Kb)"
/home/vinx/.local/bin/cutadapt -a
AGATCGGAAGAGCACACGTCTGAACTCCAGTCA -q 20,20 -j 1 R1_5M.fastq >
SE1_trim.fastq 2>cutadaptSE1.txt
$ /usr/bin/time -f "%E time_elapsed\n%S time_sys\n%U
time_user\n%P perc_cpu\n%M mem_max(Kb)"
/home/vinx/.local/bin/cutadapt -a
AGATCGGAAGAGCACACGTCTGAACTCCAGTCA -q 20,20 -j 4 R1_5M.fastq >
SE2_trim.fastq 2>cutadaptSE2.txt
$ /usr/bin/time -f "%E time_elapsed\n%S time_sys\n%U
time_user\n%P perc_cpu\n%M mem_max(Kb)"
/home/vinx/.local/bin/cutadapt -a
AGATCGGAAGAGCACACGTCTGAACTCCAGTCA -A
AGATCGGAAGAGCGTCGTGTAGGGAAAGAGTGT -q 20,20 -j 1 R1_5M.fastq
```

```
R2_5M.fastq -o PE1_1_trim.fastq -p PE1_2_trim.fastq
2>cutadaptPE1.txt
$ /usr/bin/time -f "%E time_elapsed\n%S time_sys\n%U
time_user\n%P perc_cpu\n%M mem_max(Kb)"
/home/vinx/.local/bin/cutadapt -a
AGATCGGAAGAGCACACGTCTGAACTCCAGTCA -A
AGATCGGAAGAGCGTCGTGTAGGGAAAGAGTGT -q 20,20 -j 4 R1_5M.fastq
R2_5M.fastq -o PE2_1_trim.fastq -p PE2_2_trim.fastq
2>cutadaptPE2.txt
$ echo FASTQC
$ /usr/bin/time -f "%E time_elapsed\n%S time_sys\n%U
time_user\n%P perc_cpu\n%M mem_max(Kb)" fastqc R1_5M.fastq
2>fastqcSE1.txt
$ /usr/bin/time -f "%E time_elapsed\n%S time_sys\n%U
time_user\n%P perc_cpu\n%M mem_max(Kb)" fastqc R1_5M.fastq
R2_5M.fastq 2>fastqcPE1.txt
$ echo SOAPNUKE
$ /usr/bin/time -f "%E time_elapsed\n%S time_sys\n%U
time_user\n%P perc_cpu\n%M mem_max(Kb)" SOAPnuke filter -1
R1_5M.fastq -C SE1_trim.fastq -o resultSE1 -T 1 -l 20 -f
AGATCGGAAGAGCACACGTCTGAACTCCAGTCA 2>soapnukeSE1.txt
$ /usr/bin/time -f "%E time_elapsed\n%S time_sys\n%U
time_user\n%P perc_cpu\n%M mem_max(Kb)" SOAPnuke filter -1
R1_5M.fastq -C SE2_trim.fastq -o resultSE2 -T 4 -l 20 -f
AGATCGGAAGAGCACACGTCTGAACTCCAGTCA 2>soapnukeSE2.txt
$ /usr/bin/time -f "%E time_elapsed\n%S time_sys\n%U
time_user\n%P perc_cpu\n%M mem_max(Kb)" SOAPnuke filter -1
R1_5M.fastq -2 R2_5M.fastq -C PE1_1_trim.fastq -D
PE1_2_trim.fastq -o resultPE1 -T 1 -l 20 -f
AGATCGGAAGAGCACACGTCTGAACTCCAGTCA -r
AGATCGGAAGAGCGTCGTGTAGGGAAAGAGTGT 2>soapnukePE1.txt
$ /usr/bin/time -f "%E time_elapsed\n%S time_sys\n%U
time_user\n%P perc_cpu\n%M mem_max(Kb)" SOAPnuke filter -1
R1_5M.fastq -2 R2_5M.fastq -C PE2_1_trim.fastq -D
PE2_2_trim.fastq -o resultPE2 -T 4 -l 20 -f
AGATCGGAAGAGCACACGTCTGAACTCCAGTCA -r
AGATCGGAAGAGCGTCGTGTAGGGAAAGAGTGT 2>soapnukePE2.txt
$ echo BBDUK
$ /usr/bin/time -f "%E time_elapsed\n%S time_sys\n%U
time_user\n%P perc_cpu\n%M mem_max(Kb)" bbduk.sh in1=R1_5M.fastq
```

```
out1=SE1_trim.fastq t=1 qtrim=rl trimq=20 ktrim=r k=23 mink=11
hdist=1 literal=AGATCGGAAGAGCACACGTCTGAACTCCAGTCA 2>bbdukSE1.txt
$ /usr/bin/time -f "%E time_elapsed\n%S time_sys\n%U
time_user\n%P perc_cpu\n%M mem_max(Kb)" bbduk.sh in1=R1_5M.fastq
out1=SE2_trim.fastq t=4 qtrim=rl trimq=20 ktrim=r k=23 mink=11
hdist=1 literal=AGATCGGAAGAGCACACGTCTGAACTCCAGTCA 2>bbdukSE2.txt
$ /usr/bin/time -f "%E time_elapsed\n%S time_sys\n%U
time_user\n%P perc_cpu\n%M mem_max(Kb)" bbduk.sh in1=R1_5M.fastq
in2=R2_5M.fastq out1=PE1_1_trim.fastq out2=PE1_2_trim.fastq t=1
qtrim=rl trimq=20 ktrim=r k=23 mink=11 hdist=1
literal=AGATCGGAAGAGCACACGTCTGAACTCCAGTCA,AGATCGGAAGAGCGTCGTGTAG
GGAAAGAGTGT tpe tbo 2>bbdukPE1.txt
$ /usr/bin/time -f "%E time_elapsed\n%S time_sys\n%U
time_user\n%P perc_cpu\n%M mem_max(Kb)" bbduk.sh in1=R1_5M.fastq
in2=R2_5M.fastq out1=PE2_1_trim.fastq out2=PE2_2_trim.fastq t=4
qtrim=rl trimq=20 ktrim=r k=23 mink=11 hdist=1
literal=AGATCGGAAGAGCACACGTCTGAACTCCAGTCA,AGATCGGAAGAGCGTCGTGTAG
GGAAAGAGTGT tpe tbo 2>bbdukPE2.txt
$ echo TRIMMOMATIC
$ /usr/bin/time -f "%E time_elapsed\n%S time_sys\n%U
time_user\n%P perc_cpu\n%M mem_max(Kb)" trimmomatic SE -threads
1 -phred33 R1_5M.fastq SE1_trim.fastq
ILLUMINACLIP:adapterSE.fa:2:30:10 SLIDINGWINDOW:4:20
2>trimmomaticSE1.txt
$ /usr/bin/time -f "%E time_elapsed\n%S time_sys\n%U
time_user\n%P perc_cpu\n%M mem_max(Kb)" trimmomatic SE -threads
4 -phred33 R1_5M.fastq SE2_trim.fastq
ILLUMINACLIP:adapterSE.fa:2:30:10 SLIDINGWINDOW:4:20
2>trimmomaticSE2.txt
$ /usr/bin/time -f "%E time_elapsed\n%S time_sys\n%U
time_user\n%P perc_cpu\n%M mem_max(Kb)" trimmomatic PE -threads
1 -phred33 R1_5M.fastq R2_5M.fastq PE1_1_trim.fastq
PE1_1_un_trim.fastq PE1_2_trim.fastq PE1_2_un_trim.fastq
ILLUMINACLIP:adapterPE.fa:2:30:10 SLIDINGWINDOW:4:20
2>trimmomaticPE1.txt
$ /usr/bin/time -f "%E time_elapsed\n%S time_sys\n%U
time_user\n%P perc_cpu\n%M mem_max(Kb)" trimmomatic PE -threads
4 -phred33 R1_5M.fastq R2_5M.fastq PE2_1_trim.fastq
PE2_1_un_trim.fastq PE2_2_trim.fastq PE2_2_un_trim.fastq
```

```
ILLUMINACLIP:adapterPE.fa:2:30:10 SLIDINGWINDOW:4:20
2>trimmomaticPE2.txt
```

SOAPnuke (Chen et al., 2018b) was discarded from the benchmark for producing outputs with a different number of reads when the only argument changed was the number of cores used. AfterQC (Chen et al., 2017) was discarded because fastp was developed as a faster alternative to it, and we confirmed this by comparing the elapsed time during the preprocessing. FastQC is used only to view the quality control report, being benchmarked to serve as a reference for the time needed to build the report.

The Reference Sequence NC_000004.12 was used to create the simulated datasets for the decontamination tools benchmark. Single-end (SE) and paired-end (PE) inputs, composed by 25% (b3h1), 50% (b2h2), and 75% (b1h3) of human reads were created using InSilicoSeq:

```
$ iss generate --cpus 40 --model novaseq --genomes
hsSource.fasta --n_reads 6m --output human --quiet
$ sed 's/^@NC_/@HUMAN_NC_/g' human_R1.fastq > h1.fastq
$ nohup iss generate --cpus 40 --model novaseq -k bacteria -U
200 --n_reads 400 --output bac --quiet &
$ nohup iss generate --cpus 40 --model novaseq --genomes
bacSource.fasta --n_reads 6m --output bac --quiet &
$ sed 's/^@/@BACTERIA_/g' bac_R1.fastq > b1.fastq
$ head -n 4000000 b1.fastq > b1mh3m_1.fastq
$ head -n 8000000 b1.fastq > b2mh2m_1.fastq
$ cat b1.fastq > b3mh1m_1.fastq
$ head -n 4000000 b2.fastq > b1mh3m_2.fastq
$ head -n 8000000 b2.fastq > b2mh2m_2.fastq
$ cat b2.fastq > b3mh1m_2.fastq
$ head -n 4000000 h1.fastq >> b3mh1m_1.fastq
$ head -n 8000000 h1.fastq >> b2mh2m_1.fastq
$ cat h1.fastq >> b1mh3m_1.fastq
$ head -n 4000000 h2.fastq >> b3mh1m_2.fastq
$ head -n 8000000 h2.fastq >> b2mh2m_2.fastq
$ cat h2.fastq >> b1mh3m_2.fastq
```

And then processed by the following script:

```
$ #!/bin/bash
$ path="."
$ files=$(ls $path | grep _1.fastq$)
```

```
$ ref="Homo_sapiens.GRCh38.dna.primary_assembly.fa"
$ echo BOWTIE2
$ bowtie2-build $ref bt2idx/hs102 -p 8
$ echo BWA-MEM
$ bwa index $ref
$ echo BBMAP
$ bbmap.sh t=8 -Xmx24g ref="$ref"
$ echo HISAT2
$ hisat2-build $ref bt2idx/hs102 -p 8
$ hisat2_extract_splice_sites.py Homo_sapiens.GRCh38.102.gtf >
hsaGtfR102.txt
$ for i in $files
$ do
$     temp=${i%_1.fastq}
$     echo $temp
$     echo BOWTIE2
$     echo SE
$     /usr/bin/time -f "%E time_elapsed\n%S time_sys\n%U
time_user\n%P perc_cpu\n%M mem_max(Kb)" bowtie2 -x bt2idx/hs102
-U $i -S SAM/"$temp"_bt2SE.sam -p 4
$     echo PE
$     /usr/bin/time -f "%E time_elapsed\n%S time_sys\n%U
time_user\n%P perc_cpu\n%M mem_max(Kb)" bowtie2 -x bt2idx/hs102
-1 $i -2 "$temp"_2.fastq -S SAM/"$temp"_bt2PE.sam -p 4
$     echo BWA-MEM
$     echo SE
$     /usr/bin/time -f "%E time_elapsed\n%S time_sys\n%U
time_user\n%P perc_cpu\n%M mem_max(Kb)" bwa mem $ref -t 4 $i >
SAM/"$temp"_bwaSE.sam
$     echo PE
$     /usr/bin/time -f "%E time_elapsed\n%S time_sys\n%U
time_user\n%P perc_cpu\n%M mem_max(Kb)" bwa mem $ref -t 4 $i
"$temp"_2.fastq > SAM/"$temp"_bwaPE.sam
$     echo BBMAP
$     echo SE
$     /usr/bin/time -f "%E time_elapsed\n%S time_sys\n%U
time_user\n%P perc_cpu\n%M mem_max(Kb)" bbmap.sh t=4 -Xmx24g
in="$i" out="SAM/"$temp"_bbmapSE.sam"
$     echo PE
```

```
$    /usr/bin/time -f "%E time_elapsed\n%S time_sys\n%U
time_user\n%P perc_cpu\n%M mem_max(Kb)" bbmap.sh t=4 -Xmx24g
in1="$i" in2="$temp"_2.fastq out="SAM/"$temp"_bbmapPE.sam"
$    echo HISAT2
$    echo SE
$    /usr/bin/time -f "%E time_elapsed\n%S time_sys\n%U
time_user\n%P perc_cpu\n%M mem_max(Kb)" hisat2 -x bt2idx/hs102 -
-known-splicesite-infile hsaGtfR102.txt -U $i -S
SAM/"$temp"_ht2SE.sam -p 4
$    echo PE
$    /usr/bin/time -f "%E time_elapsed\n%S time_sys\n%U
time_user\n%P perc_cpu\n%M mem_max(Kb)" hisat2 -x bt2idx/hs102 -
-known-splicesite-infile hsaGtfR102.txt -1 $i -2 "$temp"_2.fastq
-S SAM/"$temp"_ht2PE.sam -p 4
$ done
```

The aligned and unaligned reads were extracted:

```
$ #!/bin/bash
$ path="SAM/"
$ files=$(ls $path)
$ for i in $files
$ do
$    temp=${i%.sam}
$    echo $temp
$    samtools view -bS "$path$i" > "$path$temp".bam
$    samtools view -b -f 4 "$path$temp".bam >
"$path$temp".bam_unaligned.bam
$    samtools sort -n "$path$temp".bam_unaligned.bam -o
"$path$temp".bam_unaligned_sorted.bam
$    samtools bam2fq "$path$temp".bam_unaligned_sorted.bam >
"$path$temp".bam_unaligned.fastq
$    samtools view -b -F 4 "$path$temp".bam >
"$path$temp".bam_aligned.bam
$    samtools sort -n "$path$temp".bam_aligned.bam -o
"$path$temp".bam_aligned_sorted.bam
$    samtools bam2fq "$path$temp".bam_aligned_sorted.bam >
"$path$temp".bam_aligned.fastq
$ done
```

And the four confusion matrix categories were counted:

```
$ #!/bin/bash
$ path="SAM/"
$ files=$(ls $path | grep d.fastq$)
$ for i in $files
$ do
$     temp=${i%.fastq}
$     echo $temp
$     ((nreads=$(wc -l < "$path$i") / 4 ))
$     echo "Number of reads: " $nreads
$     nB=$(grep -c '^@BACTERIA' "$path$i")
$     nH=$(grep -c '^@HUMAN_NC' "$path$i")
$     echo "Number of human reads: " $nH
$     echo "Number of non-human reads: " $nB
$     echo ""
$ done
```

Producing an output like:

b1h3.bam_aligned
Number of reads: 2992585
Number of human reads: 2992585
Number of non-human reads: 0

b1h3.bam_unaligned
Number of reads: 1007415
Number of human reads: 7415
Number of human reads: 1000000

A recent study also benchmarked tools for decontamination (Czajkowski et al., 2019), with Bowtie2 outperforming the other tools.

For the taxonomic benchmark, we used the NCBI-nr protein file to build the DIAMOND, Kraken2 (Wood et al., 2019), and Kaiju indices. The indices were built using the same input for a fair comparison. Kaiju and Kraken2 perform fast taxonomic classifications using sequences as inputs, while BASTA (Kahlke & Ralph, 2019) and Krona (Ondov et al., 2011) require alignment results. Krona is mainly used to view the taxonomic results, but as it can classify BLAST/DIAMOND outputs, we benchmarked it as a reference for the MCC metric.

Building the indices and performing the analyses on the simulated dataset 1:

```
$ echo DIAMOND
$ diamond makedb --in nr -d diamond/nr160121
$ /usr/bin/time -f "%E time_elapsed\n%S time_sys\n%U
time_user\n%P perc_cpu\n%M mem_max(Kb)" diamond blastx -d
diamond/nr160121 -q sim1.fastq -o results/sim1.m8 --top 3 --
threads 64 --more-sensitive
$ echo KAIJU
$ kaiju-convertNR -t nodes.dmp -g prot.accession2taxid -e
~/miniconda3/envs/medusaPipeline/bin/kaiju-excluded-
accessions.txt -a -o kaijuNR.fasta -i ../../alignment/db/nr
$ kaiju-mkbwt -n 8 -a ACDEFGHIKLMNPQRSTVWY -o kaijuNR
kaijuNR.fasta
$ kaiju-mkfmi kaijuNR
$ /usr/bin/time -f "%E time_elapsed\n%S time_sys\n%U
time_user\n%P perc_cpu\n%M mem_max(Kb)" kaiju -t nodes.dmp -f
kaijuNR.fmi -i sim1.fastq -o results/sim1kaiju.out -z 64
$ kaiju-addTaxonNames -t nodes.dmp -n names.dmp -r
superkingdom,phylum,class,order,family,genus,species -i
results/sim1kaiju.out -o results/sim1kaiju_names.out
$ echo KRAKEN2
$ kraken2-build --download-taxonomy --db kraken2/ --threads 20
$ kraken2-build --download-library nr --db kraken2/ --protein --
threads 20
$ kraken2-build --build --db kraken2/ --protein --threads 20
$ kraken2-build --clean --db kraken2/
$ /usr/bin/time -f "%E time_elapsed\n%S time_sys\n%U
time_user\n%P perc_cpu\n%M mem_max(Kb)" kraken2 --db kraken2/ --
threads 64 --use-names --report results/sim1k2report.txt
sim1.fastq > results/sim1k2.out
$ echo KRONA
$ ~/miniconda3/envs/medusaPipeline/opt/krona/updateAccessions.sh
$ ~/miniconda3/envs/medusaPipeline/opt/krona/updateTaxonomy.sh
$ /usr/bin/time -f "%E time_elapsed\n%S time_sys\n%U
time_user\n%P perc_cpu\n%M mem_max(Kb)" ktClassifyBLAST
results/sim1.m8 -o results/sim1krona.txt
$ echo BASTA
$ basta taxonomy
$ basta download prot
```

```
$ /usr/bin/time -f "%E time_elapsed\n%S time_sys\n%U
time_user\n%P perc_cpu\n%M mem_max(Kb)" basta sequence
results/sim1.m8 results/basta_out.txt prot -l 1 -m 1 -b True
```

BASTA's run was aborted after 20 days. For this reason, BASTA is not present in Supplementary Figure S4 panels (A), (B), and (D). Kraken2 classified only 2129 (0.42%) sequences from 507429. We built again the Kraken2 index, without the cleaning call to maintain all files, and noticed that only a few identifiers from NCBI-nr were mapped. Then, we tried the fix_unmapped script from Kraken tools, as suggested by Jennifer Lu. Although the new Kraken2 index correctly maps the identifiers, no sequences (0%) were classified after this fix.

Tools for metagenomic assembly were extensively benchmarked by CAMI (Critical Assessment of Metagenome Interpretation), using gold-standard assemblies and MetaQUAST (Mikheenko et al., 2016) to assess the results. The CAMI benchmarks show that the performances of MEGAHIT (Li et al., 2016) and MetaSPAdes (Nurk et al., 2017) are quite similar (Meyer et al., 2021). We tested MetaSpades 3.13.2 and 3.15.2, but both versions failed to complete the assemble due to memory issues. MetaSpades 3.13.2 failed due to a segmentation fault in every test, and version 3.15.2 was unable to allocate the required memory. As MEGAHIT 1.2.9 finished the tests successfully, it was chosen as the pipeline assembler.

References

Araujo, F. A. et al. (2018). GO FEAT: A rapid web-based functional annotation tool for genomic and transcriptomic data. Scientific Reports. doi:10.1038/s41598-018-20211-9

BBTools. http://jgi.doe.gov/data-and-tools/bb-tools/. Accessed 07 Oct 2021.

Bolger, A. M., Lohse, M., & Usadel, B. (2014). Trimmomatic: a flexible trimmer for Illumina sequence data. Bioinformatics. doi:10.1093/bioinformatics/btu170

Breitwieser, F. P., Lu, J., & Salzberg, S. L. (2019). A review of methods and databases for metagenomic classification and assembly. Briefings in Bioinformatics. doi:10.1093/bib/bbx120

Buchfink, B., Xie, C., & Huson, D. H. (2015). Fast and sensitive protein alignment using DIAMOND. Nature Methods. doi:10.1038/nmeth.3176

Chen, S. et al. (2017). AfterQC: Automatic filtering, trimming, error removing and quality control for fastq data. BMC Bioinformatics. doi: 10.1186/s12859-017-1469-3

Chen, S. et al. (2018a). Fastp: An ultra-fast all-in-one FASTQ preprocessor. Bioinformatics. doi:10.1093/bioinformatics/bty560

Chen, Y. et al. (2018b). SOAPnuke: a MapReduce acceleration-supported software for integrated quality control and preprocessing of high-throughput sequencing data. Gigascience. doi:10.1093/gigascience/gix120

Chicco, D., & Jurman, G. (2020). The advantages of the Matthews correlation coefficient (MCC) over F1 score and accuracy in binary classification evaluation. BMC Genomics. doi:10.1186/s12864-019-6413-7

Clarke, E. L. et al. (2019). Sunbeam: An extensible pipeline for analyzing metagenomic sequencing experiments. Microbiome. doi:10.1186/s40168-019-0658-x

Czajkowski, M. D. et al. (2019). GencOf: A graphical user interface to rapidly remove human genome contaminants from metagenomic datasets. Bioinformatics. doi:10.1093/bioinformatics/bty963

Dale, R. et al. (2018). Bioconda: Sustainable and comprehensive software distribution for the life sciences. Nature Methods. doi:10.1038/s41592-018-0046-7

Dong, X., & Strous, M. (2019). An Integrated Pipeline for Annotation and Visualization of Metagenomic Contigs. Frontiers in Genetics. doi:10.3389/fgene.2019.00999

FastQC. https://www.bioinformatics.babraham.ac.uk/projects/fastqc/. Accessed 07 Oct 2021.

Franzosa, E. A. et al. (2018). Species-level functional profiling of metagenomes and metatranscriptomes. Nature Methods. doi:10.1038/s41592-018-0176-y

Gourlé, H. et al. (2019). Simulating Illumina metagenomic data with InSilicoSeq. Bioinformatics. doi:10.1093/bioinformatics/bty630

HMMER 3. http://hmmer.org/. Accessed 07 Oct 2021.

Huerta-Cepas, J. et al. (2017). Fast genome-wide functional annotation through orthology assignment by eggNOG-mapper. Molecular Biology and Evolution. doi:10.1093/molbev/msx148

Huson, D. H. et al. (2016). MEGAN Community Edition - Interactive Exploration and Analysis of Large-Scale Microbiome Sequencing Data. PLoS Computational Biology. doi:10.1371/journal.pcbi.1004957

Kahlke, T., & Ralph, P. J. (2019). BASTA - Taxonomic classification of sequences and sequence bins using last common ancestor estimations. Methods in Ecology and Evolution. doi:10.1111/2041-210X.13095

Köster, J., & Rahmann, S. (2012). Snakemake-a scalable bioinformatics workflow engine. Bioinformatics. doi:10.1093/bioinformatics/bts480

Langmead, B., & Salzberg, S. L. (2012). Fast gapped-read alignment with Bowtie 2. Nature Methods. doi:10.1038/nmeth.1923

Li, D. et al. (2016). MEGAHIT v1.0: A fast and scalable metagenome assembler driven by advanced methodologies and community practices. Methods. doi:10.1016/j.ymeth.2016.02.020

Li, H. et al. (2009). The Sequence Alignment/Map format and SAMtools. Bioinformatics. doi:10.1093/bioinformatics/btp352

Li, H., & Durbin, R. (2009). Fast and accurate short read alignment with Burrows-Wheeler transform. Bioinformatics. doi:10.1093/bioinformatics/btp324

Lindgreen, S., Adair, K. L., & Gardner, P. P. (2016). An evaluation of the accuracy and speed of metagenome analysis tools. Scientific Reports. doi:10.1038/srep19233

Martin, M. (2011). Cutadapt removes adapter sequences from high-throughput sequencing reads. EMBnet.journal. doi:10.14806/ej.17.1.200

Menzel, P., Ng, K. L., & Krogh, A. (2016). Fast and sensitive taxonomic classification for metagenomics with Kaiju. Nature Communications. doi:10.1038/ncomms11257

Meyer, F. et al. (2021). Tutorial: assessing metagenomics software with the CAMI benchmarking toolkit. Nature Protocols. doi:10.1038/s41596-020-00480-3

Mikheenko, A., Saveliev, V., & Gurevich, A. (2016). MetaQUAST: Evaluation of metagenome assemblies. Bioinformatics. doi:10.1093/bioinformatics/btv697

Nurk, S., Meleshko, D., Korobeynikov, A., & Pevzner, P. A. (2017). MetaSPAdes: A new versatile metagenomic assembler. Genome Research. doi:10.1101/gr.213959.116

Ondov, B. D., Bergman, N. H., & Phillippy, A. M. (2011). Interactive metagenomic visualization in a Web browser. BMC Bioinformatics. doi:10.1186/1471-2105-12-385

Pedersen, M. W. et al. (2015). Ancient and modern environmental DNA. Philosophical Transactions of the Royal Society B. doi:10.1098/rstb.2013.0383

Pertea, M. et al. (2016). Transcript-level expression analysis of RNA-seq experiments with HISAT, StringTie and Ballgown. Nature Protocols. doi:10.1038/nprot.2016.095

Westreich, S. T. et al. (2018). SAMSA2: A standalone metatranscriptome analysis pipeline. BMC Bioinformatics. doi:10.1186/s12859-018-2189-z

Wood, D. E., Lu, J., & Langmead, B. (2019). Improved metagenomic analysis with Kraken 2. Genome Biology. doi:10.1186/s13059-019-1891-0

Zhang, J. et al. (2014). PEAR: A fast and accurate Illumina Paired-End reAd mergeR. Bioinformatics. doi:10.1093/bioinformatics/btt593
